# Retinoic Acid Signaling Regulates Differential Expression of the Tandemly-Duplicated Long Wavelength-Sensitive Cone Opsin Genes in Zebrafish

**DOI:** 10.1371/journal.pgen.1005483

**Published:** 2015-08-21

**Authors:** Diana M. Mitchell, Craig B. Stevens, Ruth A. Frey, Samuel S. Hunter, Ryuichi Ashino, Shoji Kawamura, Deborah L. Stenkamp

**Affiliations:** 1 Department of Biological Sciences, University of Idaho, Moscow, Idaho, United States of America; 2 Bioinformatics and Computational Biology Graduate Program, University of Idaho, Moscow, Idaho, United States of America; 3 Department of Integrated Biosciences, Graduate School of Frontier Sciences, The University of Tokyo, Kashiwa, Chiba, Japan; 4 Neuroscience Graduate Program, University of Idaho, Moscow, Idaho, United States of America; New York University, UNITED STATES

## Abstract

The signaling molecule retinoic acid (RA) regulates rod and cone photoreceptor fate, differentiation, and survival. Here we elucidate the role of RA in differential regulation of the tandemly-duplicated *long wavelength-sensitive* (*LWS*) cone opsin genes. Zebrafish embryos were treated with RA from 48 hours post-fertilization (hpf) to 75 hpf, and RNA was isolated from eyes for microarray analysis. ~170 genes showed significantly altered expression, including several transcription factors and components of cellular signaling pathways. Of interest, the *LWS1* opsin gene was strongly upregulated by RA. *LWS1* is the upstream member of the tandemly duplicated *LWS* opsin array and is normally not expressed embryonically. Embryos treated with RA 48 hpf to 100 hpf or beyond showed significant reductions in *LWS2*-expressing cones in favor of *LWS1*-expressing cones. The *LWS* reporter line, *LWS-PAC(H)* provided evidence that individual LWS cones switched from *LWS2* to *LWS1* expression in response to RA. The RA signaling reporter line, *RARE*:*YFP* indicated that increased RA signaling in cones was associated with this opsin switch, and experimental reduction of RA signaling in larvae at the normal time of onset of *LWS1* expression significantly inhibited *LWS1* expression. A role for endogenous RA signaling in regulating differential expression of the *LWS* genes in postmitotic cones was further supported by the presence of an RA signaling domain in ventral retina of juvenile zebrafish that coincided with a ventral zone of *LWS1* expression. This is the first evidence that an extracellular signal may regulate differential expression of opsin genes in a tandemly duplicated array.

## Author Summary

Tandemly-replicated opsin genes are found in genomes of humans and zebrafish. In humans, the tandemly-replicated long wavelength-sensitive/medium wavelength-sensitive (*LWS/MWS*) array underlies trichromatic color vision; defects in these genes result in color blindness and X-linked retinal degenerations. The current model for regulation of tandemly replicated opsin genes states that stochastic interactions between upstream enhancer regions and gene promoters result in a preferential association with the *LWS* or *MWS* promoter. Here we provide evidence, from the *LWS1/LWS2* array in zebrafish, that a trans-regulatory mechanism may instead control expression. This array is orthologous to the human *LWS/MWS* array but arose through an independent gene duplication event. We identified genes that were differentially expressed in zebrafish embryo eyes in response to treatment with the developmental signaling molecule retinoic acid (RA) during photoreceptor differentiation. *LWS1* was significantly upregulated by this treatment, and we demonstrate that individual cone photoreceptors were induced by RA to switch expression from *LWS2* to *LWS1*. Experimental reduction of RA signaling inhibited expression of *LWS1*, and endogenous RA signaling domains spatially coincided with a zone of *LWS1* expression in individual cones during zebrafish retinal growth. Our findings suggest that RA signaling within the retina regulates differential expression of the *LWS* genes, and therefore that tandemly-replicated opsin genes may be amenable to therapeutic manipulation.

## Introduction

Color vision in vertebrates requires the differentiation of multiple types of cone photoreceptors in the retina, each of which has a different spectral sensitivity. Spectral sensitivities are conferred by the visual pigment present within cone photoreceptors; these pigments consist of a specific opsin protein, and a light-sensitive chromophore (11-cis retinal or 11-cis 3,4-dehydroretinal) [1]. There are five major types of photoreceptor opsins in vertebrates: RH1 (rod opsin), SWS1 (short wavelength-sensitive; blue or UV), SWS2 (short wavelength-sensitive; blue), RH2 (middle wavelength-sensitive; green), and M/LWS (middle to long wavelength-sensitive, green or red) [2]. The human retina contains rods, which express rod opsin, as well as cones, which express one of three types of cone opsin, SWS1 (blue), MWS (green), and LWS (red). In the zebrafish, by comparison, the retina contains rods as well as four major classes of cones, SWS1 (UV), SWS2 (blue), RH2 (green), and LWS (red). Individual RH2 cones may express one of four RH2 opsins, while individual LWS cones may express one of two LWS opsins [3]. In humans, the *LWS* and *MWS* opsin genes are arrayed in a tail to head manner on the X chromosome, the consequence of tandem gene duplication and evolutionary neofunctionalization [4]. Similarly, the four *RH2* genes and two *LWS* genes in zebrafish reside in independent tandem arrays [3]. The zebrafish *LWS1* gene is orthologous to the human *LWS* gene, but the *LWS/MWS* gene duplication in humans is independent from the *LWS1/LWS2* duplication in zebrafish [3].

In both mammals and teleost fish, cone photoreceptors are patterned across the retinal hemisphere with both short-range (mosaic) spacing attributes, and long-range patterns as a function of retinal eccentricity or dorsal-ventral position. For example, mouse cones show a regular local spacing [5], and pronounced dorsal-ventral gradients of *MWS* and *SWS1* opsin expression [6,7]. Human SWS1 cones also display regular local spacing [8], and the LWS and MWS cones show a central-to-peripheral gradient in density, as well as in LWS:MWS ratio, with an LWS:MWS ratio lower in central retina than in peripheral retina [9]. In zebrafish retina, on a local scale cones display a geometrically precise “row” mosaic [10,11], but cones expressing opsin genes from tandemly-replicated arrays also show larger-scale patterns. Within the central retina, RH2 cones express *RH2-1* or *RH2-2*, and LWS cones express *LWS2*; within ventral and peripheral retina the RH2 cones express *RH2-3* or *RH2-4*, and the LWS cones express *LWS1* [12].

Regulatory mechanisms for achieving photoreceptor diversity and fates are the focus of intense investigation due to their potential applications in treating retinal diseases. A transcriptional regulation model is emerging, in which the photoreceptor transcription factors Crx, NeuroD, and Rx/Rax promote photoreceptor-specific gene expression [13], and the transcription factors Nr2e3 and Nrl promote rod-specific gene expression and suppress S cone development [14,15]. The nuclear hormone receptors RXRγ and TRβ2, together with thyroid hormone (T3) promote the differentiation of MWS/LWS cones and suppress SWS1 cone opsin expression [6,16,17]. In the zebrafish, the transcription factor tbx2b supports the SWS1 (UV-sensitive) cone fate while suppressing the rod fate [18]. Also in the zebrafish, RA and the receptor RARαb favor rod fates over cone fates during progenitor proliferation [19]. However, the choice of opsin from a tandem array, such as the human *LWS/MWS* array, is thought to be a stochastic event. In the current model, during cone differentiation, an upstream locus control region (LCR) becomes preferentially associated with the *LWS* or *MWS* promoter. This association then becomes permanent, resulting in each cone expressing only one of the opsin genes of the array [20]. However, this stochastic model does not explain the spatial gradient of the LWS:MWS ratio [9], and does not explain a predicted genetic factor that influences this ratio [21].

Key signaling molecules known to be present in specific spatial gradients during vertebrate retinal development include the nuclear hormone receptor ligands retinoic acid (RA) and thyroid hormone (tri-iodothyronine, T3) [16,22]. RA is the acid derivative of Vitamin A (retinol) and in vertebrate animal models is synthesized locally within the developing eye, with high concentrations ventrally, medium concentrations dorsally, and low concentrations centrally [22–25]. RA signaling via specific receptors (RARs and/or RXRs) plays many critical roles in eye development, from early eye organogenesis up to photoreceptor differentiation and survival. For example, ventral RA is necessary for optic fissure closure [26] and for the development of ventral retina in general [25]. Numerous reports have indicated that RA promotes rod determination [19], differentiation [27–31], and survival [32,33], and these findings have been translated into promising approaches for promoting rod development in retina cultures derived from human ES and iPS cells [34–36].

In one of our previous studies, we observed that treatment with all-trans RA (at-RA) over the time of photoreceptor differentiation in zebrafish promoted the differentiation of rods and red-sensitive (LWS) cones, while reducing the differentiation of blue- (SWS2) and UV- (SWS1) sensitive cones [29]. In the present study we performed microarray analysis of genes expressed in the eyes of zebrafish embryos subjected to a similar at-RA exposure regime. Our original goal was to identify genes that may mediate the effects of RA on photoreceptor differentiation, and the microarray experiment indeed revealed numerous candidates, including components of Wnt and Bmp signaling pathways, the RA receptor RXRγa, and several additional retinal transcription factors. Of particular interest however, the microarray, along with qCPR, *in situ* hybridization studies, and the use of a transgenic reporter line, revealed that exogenous RA strongly upregulated specifically the first member of the *LWS* array, *LWS1*, while downregulating *LWS2*, within individual LWS cones. Experimental reduction of RA signaling correspondingly prevented upregulation of *LWS1*, and native RA signaling domains coincided with an endogenous *LWS1* expression zone within growing juvenile retinas. These results are the first to provide evidence for a trans-regulatory mechanism for the control of differential expression of tandemly replicated opsin genes.

## Results

### Microarray identification of RA-responsive genes in the zebrafish eye

**Fig 1:**
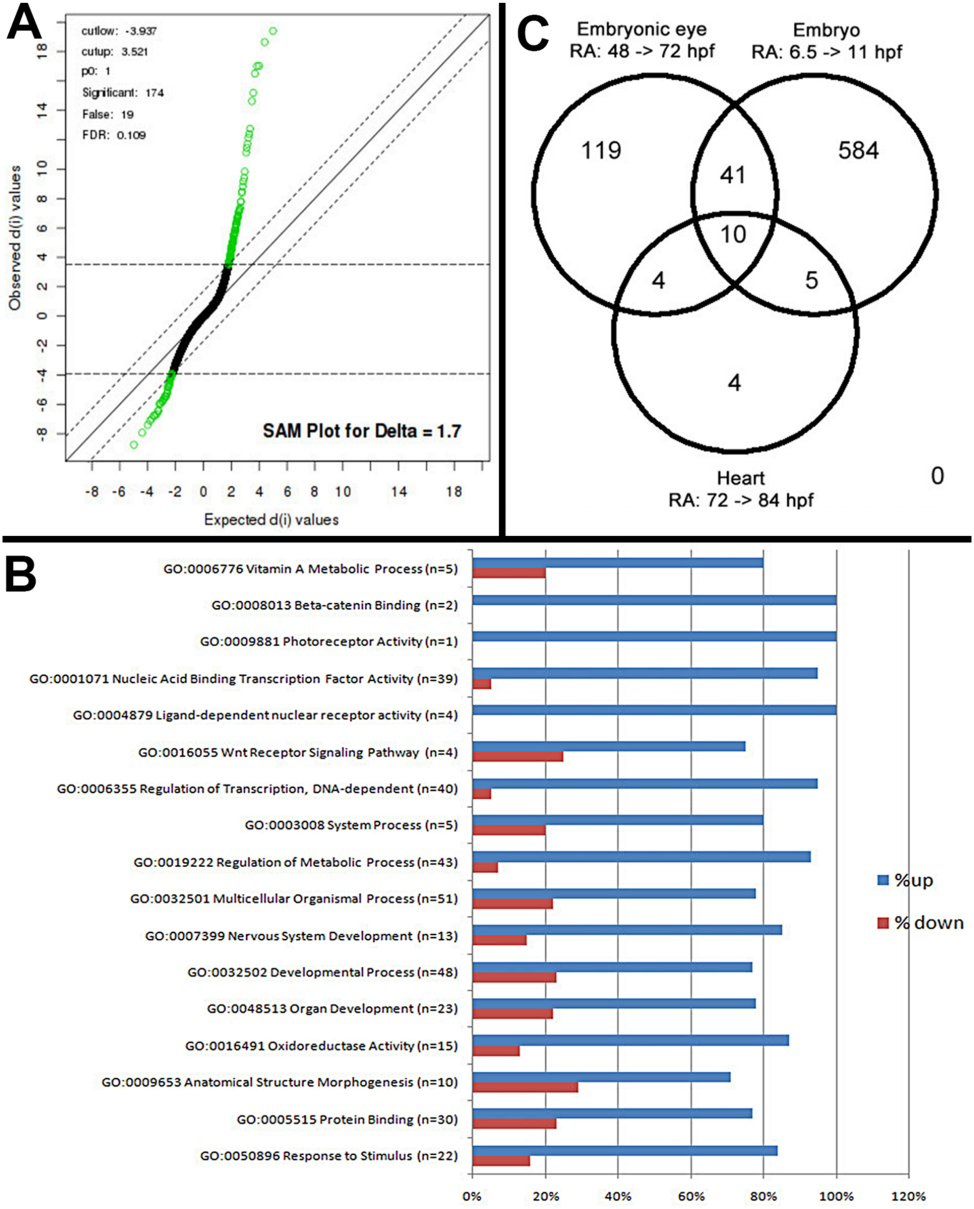
Microarray analysis of eye-specific gene expression in zebrafish embryo eyes after 48–75 hpf exposure to 0.3 μM all-trans RA. A. Statistical Analysis of Microarray (SAM) plot, at a 0.109 false discovery rate (FDR). B. Gene ontology (GO) analysis; selected GO categories shown. C. Venn diagram comparing differentially expressed genes in eyes of RA-treated embryos with those of RA-treated whole embryos during somitogenesis and with those of hearts of RA-treated larvae.

An objective of this analysis was to identify eye-specific genes that are regulated by exogenous RA in zebrafish. The zebrafish embryonic retina exists as a proliferative neuroepithelium at 24 hpf, and after 48 hpf photoreceptor differentiation occurs concomitantly with lamination of the retina. By 72 hpf the zebrafish retina has formed the neurons of the ganglion cell layer (GCL), inner nuclear layer (INL) and outer nuclear layer (ONL) [37,38]. Microarray analysis of embryonic eyes of groups treated with 0.3 μM RA or DMSO (controls) over the period of embryonic photoreceptor differentiation (from 48 hpf–75 hpf) identified a list of 174 Affymetrix probe sequences (S1 Table) that were significantly differentially expressed at the 10% FDR (Fig 1A). Of this list 63% were known while 37% represented unconfirmed, potentially novel, gene sequences. Analysis of the list of genes was performed through the use of the gene ontology clustering software GOEAST [39]. A summary of this analysis, listing specific biological and molecular process categories of interest, finds that most differentially expressed genes were upregulated by RA treatment (Fig 1B). The broader categories overrepresented in the differentially expressed list included those related to development and morphogenesis, stimulus response, and metabolic processes. The more specific categories included vitamin A metabolism, transcription factors, components of cellular signaling pathways, and surprisingly only one gene related to photoreceptor activity (Fig 1B).

#### Genes associated with vitamin A metabolism (retinoid metabolism) and retinoid signaling (Table 1)

**Table 1:** Differential expression of genes involved in RA synthesis and signaling in eyes of embryonic zebrafish treated with RA.

Gene	Log2 Fold Change	p-value	Function in developing eye	References
*aldh1a2*	-1.53	7.9E-03	RA synthesis	[23,29]
*cyp26a1*	3.68	6.4E-06	RA catabolism	[43]
*cyp26b1*	4.52	1.3E-05	RA catabolism	[45]
*dhrs3a*	3.89	3.8E-05	RA precursor catabolism	[26]
*rxrγa*	0.62	1.4E-03	RXR receptor	[19]

Exogenous RA upregulated cyp26b1 and cyp26a1, which encode enzymes that catabolize all-trans RA [40], and dhrs3a, which encodes a dehydrogenase that reduces the amount of retinaldehyde available for conversion to all-trans RA [41]. Significantly downregulated was aldh1a2, encoding a retinaldehyde dehydrogenase that converts retinaldehyde into RA [42]. At the 20% FDR, the RA receptor gene rxrγa was significantly upregulated. These results are consistent with exogenous RA regulating expression of RA metabolic enzymes in an attempt to achieve RA homeostasis [43,44].

#### Genes encoding transcription factors (Table 2)

**Table 2:** Differential expression of genes encoding transcription factors in eyes of embryonic zebrafish treated with RA.

Gene	Log2 Fold Change	p value	Function/expression in developing eye, if known	References
*bcor*	0.69	1.3E-03	Deleted in retinoblastomas	[49]
*emx2*	0.99	5.3E-04	In choroid fissure	[54]
*eomesa*	1.34	5.4E+02	In retina	(Thisse and Thisse, 2005; ZFIN)
*gata3*	2.34	7.7E-05	Lens development	[52]
*gata5*	1.49	1.3E-03	Upregulated in retinoblastomas	[50]
*hoxa4a*	2.03	7.0E-04	Not known	
*hoxb1b*	3.80	7.0E-05	Not known	
*hoxb2a*	1.55	2.9E-04	Not known	
*hoxb3a*	2.04	4.6E-04	Not known	
*hoxb5a*	5.48	6.4E-05	Not known	
*hoxb5b*	5.15	8.3E-05	Not known	
*hoxb6a*	2.52	3.3E-04	Not known	
*hoxb8a*	5.15	8.3E-05	Not known	
*hoxb8b*	2.51	1.3E-04	Not known	
*hoxb9a*	1.94	1.9E-04	Not known	
*hoxc1a*	1.20	1.1E-03	Not known	
*hoxc4a*	1.23	6.2E-04	Not known	
*hoxc5a*	1.88	5.5E-04	Not known	
*hoxd4a*	2.02	6.9E-04	Not known	
*irf9*	1.21	1.2E-03	Vascular cells	[55]
*meis4*.*1a*	1.98	3.2E-04	Not detected	[54]
*myog*	1.08	1.2E-03	Extraocular muscles	[47]
*nr0b2a*	1.22	1.3E-03	Not detected	[56]
*nr2f5*	1.62	1.8E-04	Ventral retina	[56]
*nrip1b*	0.59	9.8E-04	RGC and INL	[56]
*pea3 (etv4)*	-0.86	6.7E-04	Lens	[57]
*pitx2*	0.90	5.4E-04	Periocular mesenchyme	[26]
*prrx1a*	-1.46	1.1E-03	Not known	
*prrx1b*	-1.51	3.5E-04	Not known	
*rxrγa*	0.62	1.4E-03	Transiently in ONL	[19]
*six6a*	1.32	6.7E-04	Retinal progenitor cells	[51]
*zic3*	-1.09	7.0E-04	RGC intraretinal axon projections	[58]
*znf703*	1.01	1.4E-03	Periocular mesenchyme and choroid fissure	[26]

Eight of these genes are expressed in zebrafish embryonic neural retina (*emx2*, *eomesa*, *nr2f5*, *nrip1b*, *pea3*, *rxrγa*, *zic3*, *znf703*). Two additional genes are associated with periocular mesenchyme and its derivatives: *pitx2* and *myog*, which are both known targets of RA signaling in development of extraocular muscles [46–48]. Two genes (*bcor*, *gata5*) are associated with retinoblastoma [49,50], the homologue of *six6* is expressed in retinal progenitor cells [51], and that of *gata3* is important for lens development [52]. 19 transcription factor genes (*irf9*, *meis4*.*1a*, *nr0b2a*, *prrx1a*, *prrx1b*, and 14 *hox* genes) have not, to our knowledge, been shown to be expressed in ocular tissues. Surprisingly, a large number (14) of *hox* genes were significantly upregulated in the eyes of RA-treated embryos (Table 3). This finding suggests that either these genes are not fully silenced in eye tissues at 48–75 hpf, and/or that an activity downstream of RA signaling acts to modulate chromatin structure of the *hox* gene arrays so they can be activated [53].

**Table 3:** Differential expression of genes encoding components of cell signaling pathways in eyes of embryonic zebrafish treated with RA.

Gene	Log2 Fold Change	p value	Function/expression in developing eye, if known	References
*angptl5*	-1.78	5.1E-04	Not known	
*bmp2b*	0.475	1.4E-03	Dorsal retina initiation	[60]
*bmp4*	-0.635	8.4E-04	Dorsal retina	[61]
*dio2*	1.76	7.6E-04	Transiently in ventral retina	[62]
*dkk1b*	-0.844	1.0E-03	Lens	[63]
*epha4b*	1.34	1.1E-03	Temporal retina	[64]
*epo*	1.42	6.2E-04	Fetal retina	[65]
*igfbp1a*	1.48	6.1E-04	Posterior retina and hyaloid vasculature	(Thisse et al., 2001; ZFIN)
*igfbp2b*	-0.821	5.6E-03	Lens	[66]
*inhbaa*	-1.27	1.0E-03	Not known	
*ror2*	1.1	7.4E-04	Not known	
*sfrp1a*	1.44	6.4E-04	Dorsal retina identity	[67]
*socs3a*	1.58	5.8E-04	Retina^1^	[68]
*socs3b*	1.57	6.3E-04	Regenerating RGCs^1^	[69]
*spond2b*	-2.87	1.9E-04	Lens	[70]

^1^ An unspecified *socs3* is upregulated in adult retina in response to light damage of photoreceptors [71].

#### Genes encoding components of non-RA-related cellular signaling pathways (Table 3)

Seven of the genes differentially expressed in the eye in response to RA encode the extracellular signaling factors *bmp2b*, *bmp4*, *epha5b*, *igfbp1a*, *igfbp2b*, *epo*, *angpt15*, two encode intracellular modulators of signaling (*socs3a* and *socs3b*), and one encodes an enzymatic activator of thyroid hormone (T3; *dio2*). Two genes involved in controlling hematopoiesis, *epo* and *angpt15*, were upregulated by RA. An interesting subset of differentially expressed genes encodes modifiers of Wnt signaling (*dkk1b*, *sfrp1a*, *spond2b*). In addition, *ror2*, which encodes a transmembrane co-receptor involved in the non-canonical (Ca^2+^) Wnt pathway [59], was also upregulated. At the 20% FDR, *wnt11* was significantly upregulated.

The GO analysis of the microarray data indicated upregulation of only one gene involved in photoreceptor function, *opn1lw1*, the first member of the *long wavelength-sensitive* opsin gene array [3] (Fig 1B). However, further database searches revealed three additional genes associated with photoreceptor differentiation, as significantly upregulated (Table 4). *RXRγa* is expressed transiently within the ONL during photoreceptor differentiation in zebrafish [19], and its mouse homologue is suppresses S opsin in developing cones [6]. *Aanat2* encodes the rate-limiting enzyme involved in melatonin synthesis in photoreceptors [72], and *atp1a1*.*3* encodes the α subunit of Na+/K+ ATPase, which is enriched in photoreceptors [73,74]. Unexpectedly, rod opsin was not detected as upregulated by the microarray, even at the 20% FDR, although there is evidence supporting roles for RA in stimulating rod opsin expression and rod cell differentiation in the zebrafish, using alternative methods [19,29,30,33].

**Table 4:** Differential expression of eye and photoreceptor genes in eyes of embryonic zebrafish treated with RA.

Gene	Log2 Fold Change	P value	Function/expression in developing eye, if known	References
*aanat2*	1.41	9.4E-04	Developing photoreceptors	[75]
*atp1b1*.*b*	1.73	4.1E-04	Retina; enriched in photoreceptors	(Thisse et al., 2001; ZFIN) [73]
*cryaa*	1.21	4.6E-04	Lens	[54]
*crygm4*	1.52	2.9E-04	Adult lens	[76]
*opn1lw1*	5.27	5.8E-05	Red-sensitive cone photoreceptors in larvae and adults	[12]
*rxrγa*	0.623	1.4E-03	Transient in photoreceptor layer	[19]

### RA activates a restricted set of common genes in the developing zebrafish embryo

We next tested the hypothesis that RA signaling targets are tissue- and developmental stage-specific, by comparing our list of differentially expressed genes with that of others obtained following treatment of whole zebrafish embryos with RA [77,78]. A comparison with genes upregulated by RA during early somitogenesis (6.5 hpf–11 hpf) [78] identified 50 common probes (S2 Table and Fig 1C), including *cyp26a1*, *cyp26b1*, *dhrs3a*, *rxrga*, and *aldh1a2* [78]. The transcription factor-encoding genes *nr2f5*, *meis4*.*1a*, *nr0b2a*, *zic3*, *nrip1b*, *znf703*, *RXRγa*, and 10 common *hox* genes were all upregulated in response to RA, in both lists.

The results of the above comparison were then compared to a microarray dataset of genes differentially expressed in zebrafish larval hearts in response to RA [77]. Among all three data sets, 11 probes (eight genes) were commonly upregulated (Fig 1C and S2 Table). Of these, three are genes involved in RA metabolism (*cyp26a1*, *cyp26b1*, and *dhrs3a*), and two are orphan nuclear receptors (*nr2f5* and *nr0b2a*) that may be essential base regulators of retinoid metabolism [77]. The relatively small number of genes commonly to all three datasets indicates that the current dataset predominantly reflects eye-specific transcriptional responses to RA exposure.

### Validation of microarray results

**Fig 2:**
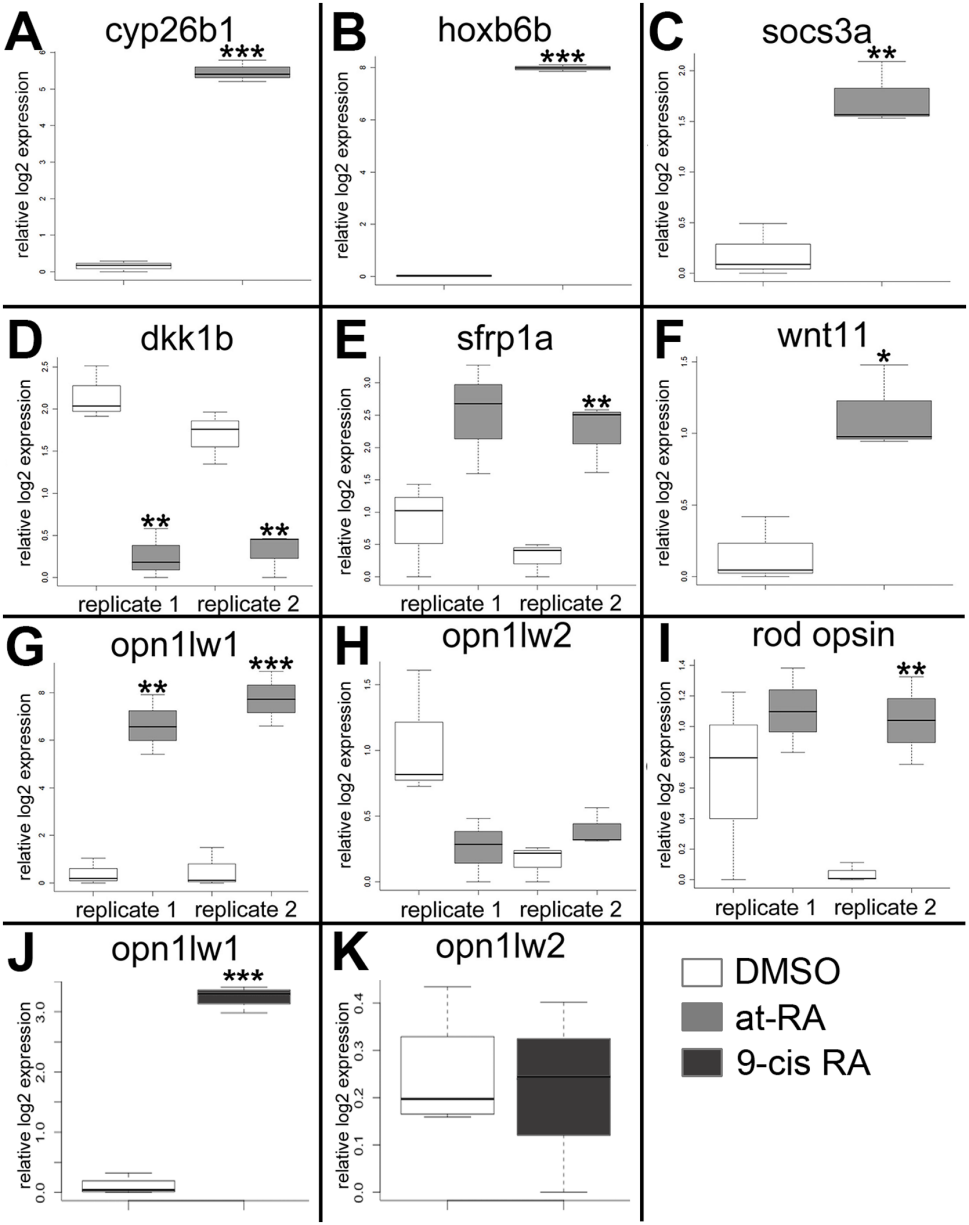
Quantitative (real-time) PCR validation of selected genes analyzed by the microarray. A. *cyp26b1*. B. *hoxb6a*. C. *socs3a*. D. *dkk1b*. E. *sfrp1a*. F. *wnt 11*. G. and J. *opn1lw1*. H. and K. *opn1lw2*. I. *rod opsin* (*RH1*). White boxes, DMSO; gray boxes, at-RA (both in embryonic eye tissues); dark gray boxes, 9-cis RA (in whole embryos). In the boxplots, the boxes demarcate the 25^th^ and 75^th^ percentiles, dark horizontal lines designate the medians, and whiskers represent the upper and lower limits. Genes in A-C, E-G, were all identified as upregulated by the microarray; *dkk1b* was identified as downregulated; *opn1w2* and *rod opsin* were not detected as differentially expressed. ***, p<0.001; **, p<0.01; *, p<0.05 (2-tailed Student’s t-test).

The following genes were selected for qPCR validation: *cyp26b1*, *hoxb6b*, *socs3a*, *dkk1b*, *sfrp1a*, *wnt11*, *opt1lw1*, *opn1lw2*, and *rho (rod opsin)* (Primer pairs are listed in Table 5). Each sample was tested with one or two separate qPCR experiments, each with three biological replicates. In brief, *dkk1* was significantly downregulated in the RA-treated samples, while *cyp26b1*, *hoxb6b*, *socs3a*, *sfrp1a*, *wnt11*, and *opn1lw1* were significantly upregulated (Fig 2A–2G), consistent with the microarray. The results of qPCR for rod opsin expression, however, were ambiguous. In one qPCR experiment this gene was found to be significantly upregulated, but not in a replicate experiment (Fig 2I). It is possible that at the selected sampling time (75 hpf), changes in rod opsin expression take place rapidly [79], and minor differences in relative embryo age in each clutch may contribute to this ambiguity. An alternative explanation is that rods make up a very small percentage of the total number of cells in the eye, and this tissue heterogeneity contributes to the inconsistency of qPCR results. However, the qPCR analyses in general confirmed differential expression where detected by microarray analysis, as well as the direction of expression changes between control and experimental groups.

**Table 5:** Primers used for q-RT-PCR.

Gene	Primer 5’ to 3’
*cyp26b1*	Forward: AGT-CCC-CGG-ACG-TTG-ACA
	Reverse: CCA-ACG-CCG-AGA-CAA-GGT
*hoxb6b*	Forward: CGC-TCG-TGC-GCT-ATT-GG
	Reverse: TCT-TGC-ACT-GGT-CCT-GGG-TTA
*socs3a*	Forward: AGG-TCA-GGG-TTT-GGT-GTG-TA
	Reverse: GAT-TTT-CTC-CCC-TCC-TGT-GT
*dkk1b*	Forward: GCC-GGT-TCT-ACG-ATG-CTC-AA
	Reverse: CCC-GCC-GCA-CCT-GAA
*wnt11*	Forward: TGA-GGA-ACC-GGC-GTT-CAG
	Reverse: CAT-TGT-TGT-GAA-GCT-GCA-TGA
*sfrp1a*	Forward: GCC-GCA-GGC-TCT-GTG-AA
	Reverse: CCG-AAT-GCT-GCC-ATG-ATG
*RH1 (rod opsin)*	Forward: CCA-ACC-GCA-GCC-ATG-AA
	Reverse: GGC-ATT-GGA-CAT-AGG-CAC-GTA
*opn1lw1 (LWS-1)*	Forward: CCC-ACA-CTG-CAT-CTC-GAC-AA
	Reverse: AAG-GTA-TTC-CCC-ATC-ACT-CCA-A
*opn1lw2 (LWS-2)*	Forward: AGA-GGG-AAG-AAC-TGG-ACT-TTC-AGA
	Reverse: TTC-AGA-GGA-GTT-TTG-CCT-ACA-TAT-GT
*18s*	Forward: GAA-CGC-CAC-TTGTCCCTCTA
	Reverse: GTT-GGT-GGA-GCG-ATT-TGT-CT
*β-actin*	Forward: GTA-CCA-CCA-GAC-AAT-ACA-GT
	Reverse: CTT-CTT-GGG-TAT-GGA-ATC-TTG-C
*nr2f5*	Forward: GAC-AGA-ATG-TTG-CCA-TGC-C
	Reverse: TCC-TGG-GCC-AAA-TTA-GCA

The very large fold-change in expression of *opn1lw1*, as detected by both microarray (S1 Table) and qPCR (Fig 2G) was rather striking. *Opn1lw1* (a.k.a. *LWS1*) encodes the first member of the tandemly-duplicated long wavelength-sensitive (*LWS*; red-sensitive) opsin array [3]. Our previous study detected increased differentiation of red-sensitive cones in response to RA treatment, with the interpretation that the increase was related to expression of the second member of the *LWS* array, *opn1lw2* (a.k.a. *LWS2*) [29], because this is the only *LWS* gene expressed in embryos [12], and because our *in situ* probes were directly complementary to this gene [3,29,80]. Therefore, we next determined whether the RA treatment affected expression of *opn1lw2/LWS2*, using qPCR. Surprisingly, levels of mRNA expression of this gene within the eye were not significantly affected by RA treatment from 48–75 hpf (Fig 2H). In separate experiments we tested the more RXR-selective retinoid, 9-cis RA, and 0.3 μM 9-cis RA treatment from 48–75 hpf significantly upregulated *LWS1* (p<0.001) in whole embryos, but did not change expression levels of *LWS2* (Fig 2J and 2K).

### Spatial distribution of opn1lw1/LWS1 and opn1lw2/LWS2 after RA exposures

**Fig 3:**
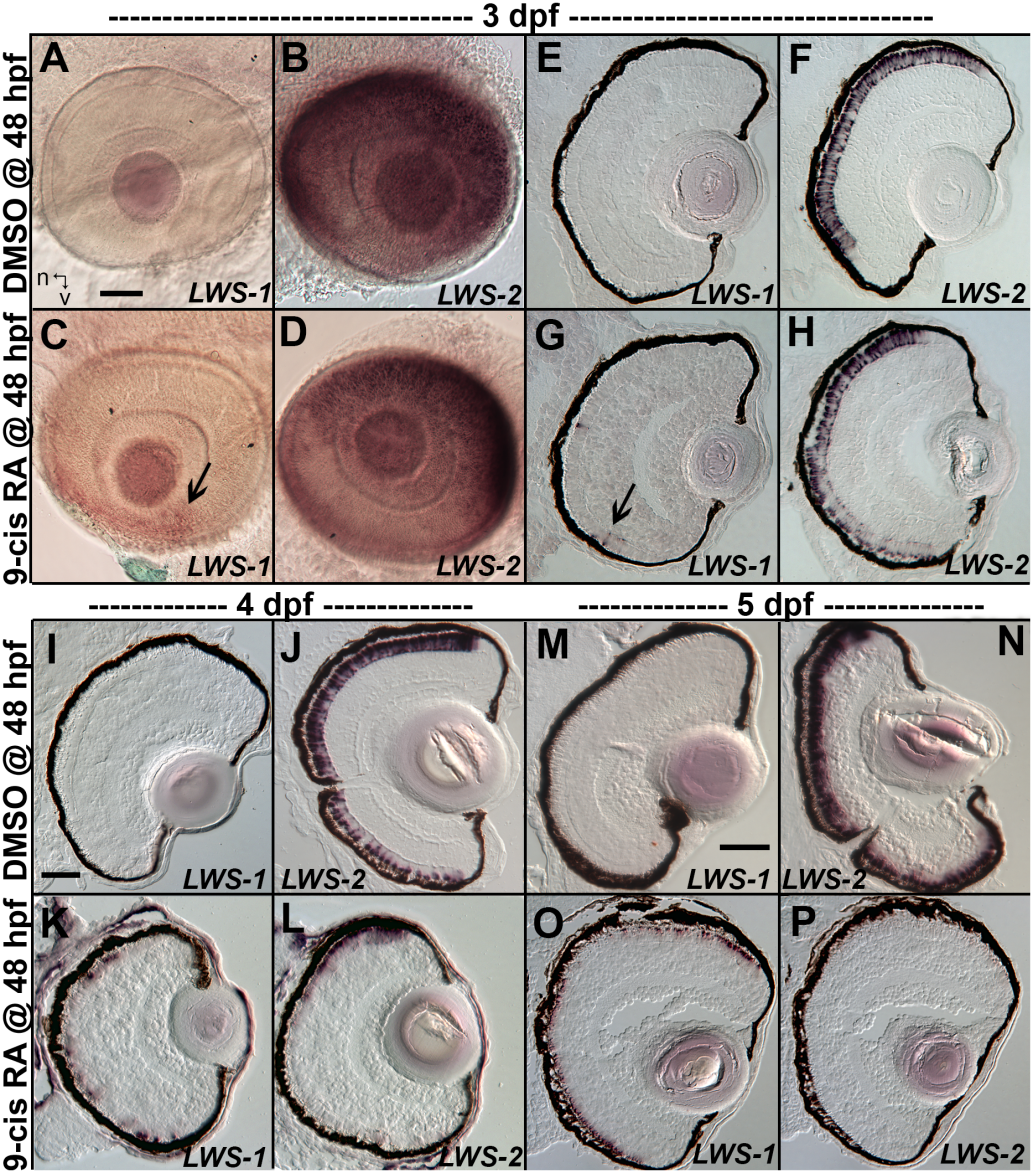
Changes in spatiotemporal patterns of expression of *LWS1* and *LWS2* in response to 9-cis RA treatment. A-H. Whole-mounted (A-D) and sectioned (E-H) embryo eyes obtained from embryos treated with DMSO (A,B,E,F) or 0.3 μM 9-cis RA (C,D,G,H) from 48 hpf to 75 hpf, and then hybridized with *LWS1* (A,E,C,G) or *LWS2* (B,G,D,H) cRNA. Arrows in C and G indicate *LWS1*-expressing cones in ventral retina; n, nasal; v, ventral. I-L. Sectioned embryo eyes obtained from embryos treated with DMSO (I,J) or 0.3 μM 9-cis RA (K,L) from 48 hpf to 4 dpf, and then hybridized with *LWS1* (I,K) or *LWS2* (J,L) cRNA. M-P. Sectioned embryo eyes obtained from embryos treated with DMSO (M,N) or 0.3 μM 9-cis RA (O,P) from 48 hpf to 4 dpf, and then hybridized with *LWS1* (M,O) or *LWS2* (N,P) cRNA. Scale bar in A (applies to A-H) = 50 um. Scale bar in I (applies to I-L) = 50 μm. Scale bar in M (applies to M-P) = 100 μm.

**Fig 4:**
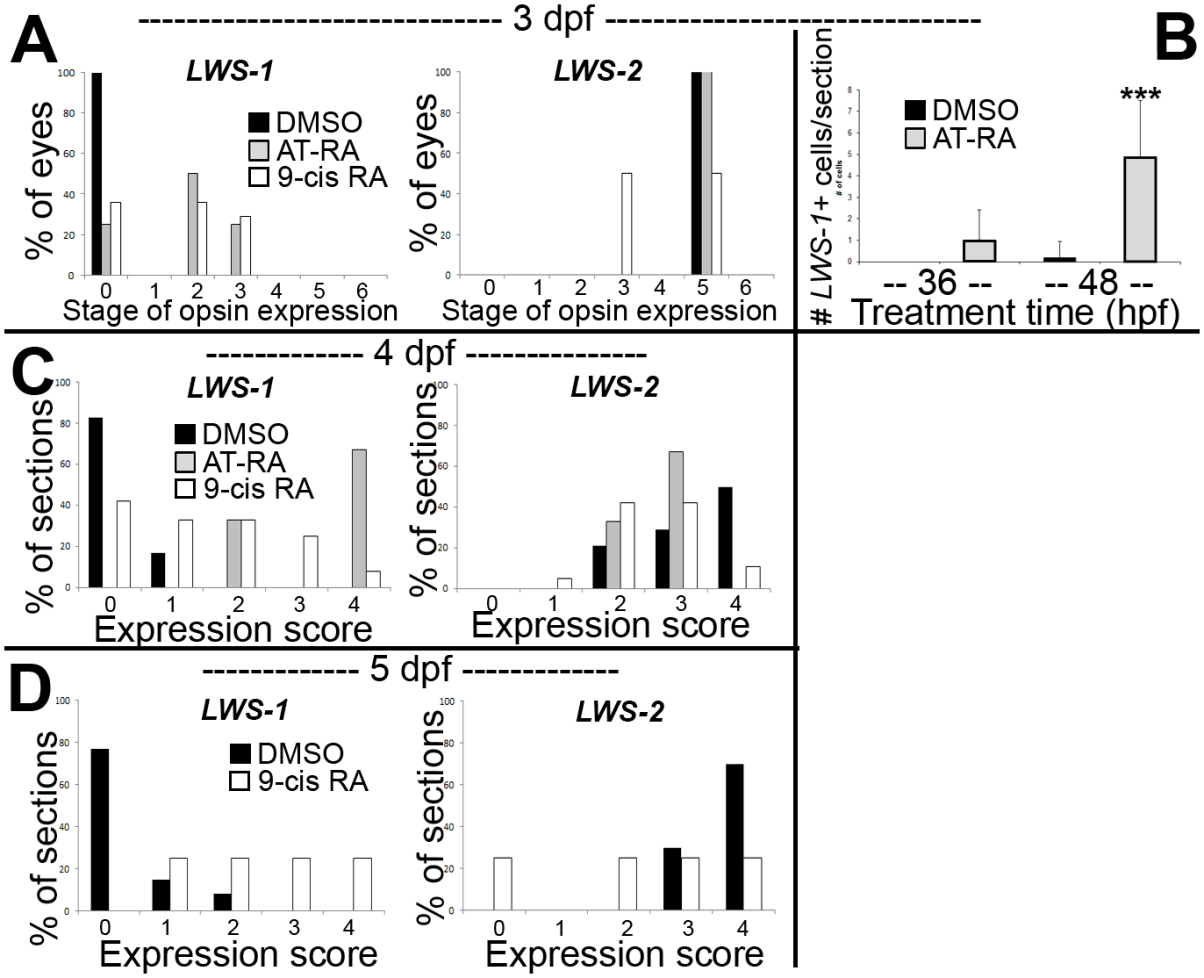
Quantitative analysis of expression patterns of *LWS1* and *LWS2* in response to all-trans RA (at-RA) or 9-cis RA treatment. A. Scoring of *LWS1* and *LWS2* expression in whole mounted eyes of embryos treated from 48 hpf to 75 hpf; stages of opsin expression are described in Materials and Methods. B. Numbers of *LWS1-* and *LWS2*-expressing cells in the outer nuclear layers of cryosections derived from embryos treated with at-RA from 36 hpf to 75 hpf as compared to treatments from 48 hpf to 75 hpf; ***, p<0.001). C. Scoring of *LWS1* and *LWS2* expression domains in the outer nuclear layers of cryosections derived from embryos treated with at-RA or 9-cis RA from 48 hpf to 4 dpf. D. Scoring of *LWS1* and *LWS2* expression domains in the outer nuclear layers of cryosections derived from embryos treated with 9-cis RA from 48 hpf to 5 dpf (C). Expression scores are described in Materials and Methods; statistical analyses (Fisher’s Exact Test) in Table 6.

When analyzed at 3 dpf (75 hpf) as whole mounts, control embryos showed widespread expression of *LWS2*, and no embryos showed expression of *LWS1* (Fig 3A and 3B), consistent with previous findings that the onset of *LWS1* expression is delayed until ~one week post-fertilization [12]. In contrast, embryos treated with at-RA or 9-cis RA still showed widespread expression of *LWS2*, but this expression occasionally appeared weaker in ventral, nasal, and/or central retina (Fig 3D). In addition, the RA-treated embryos contained *LWS1* expressing cones scattered primarily in ventral retina (Fig 3C). We scored these expression patterns, using modifications of previous criteria [79] (see Methods). This analysis revealed that eyes of RA-treated embryos reached higher stages of *LWS1* expression, but RA (either at-RA or 9-cis RA) had minimal impact on stage (distribution) of *LWS2* expression (Fig 4A and Table 6). Parallel experiments utilizing cryosections verified these findings, and demonstrated that the “RA-induced” *LWS1* expression was in the ONL (Fig 3E–3H). We quantified our findings by counting numbers of *LWS1*+ cones in each section. The statistical analysis revealed significantly greater numbers of *LWS1*+ cones in 9-cis RA treated retinas (Fig 4B). These results are consistent with those of the microarray and qPCR: an abrupt upregulation of *LWS1* in a small number of cones in RA-treated embryos as compared with essentially no expression in control embryos would be reflected by the statistically significant and high fold-change increase in levels of *LWS1* expression (S1 Table and Fig 2G), while a slight reduction in *LWS2* expression in a small number of cones may not be detected by quantitative measurements of *LWS2* mRNA expression in whole eyes (Fig 2H).

**Table 6:** Statistical evaluation of *LWS* opsin mRNA expression scoring.

Sampling Time and Method	Comparison (n)	p-value (Fisher Exact Test)	
		*LWS-1*	*LWS-2*
3 dpf wholemounts^1^	DMSO (11) vs. at-RA (4)	<0.05	n.s.^3^
	DMSO (11) vs. 9-cis RA (14)	<0.01	<0.01
4 dpf cryosections^2^	DMSO (14) vs. at-RA (3)	<0.01	n.s.
	DMSO (14) vs. 9-cis RA (19)	<0.001	<0.01
5 dpf cryosections^2^	DMSO (13) vs. 9-cis RA (4)	<0.01	<0.05

^1^ wholemounts; 7 possible scores (stages 0–6).

^2^ cryosections; 5 possible scores (stages 0–4).

^3^ n.s., not significant.

We previously demonstrated that when RA exposure takes place beginning at 36 hpf, well prior to photoreceptor terminal mitoses, a rod fate is favored over cone fates [19]. To determine whether this earlier increase in RA signaling also favors *LWS1* expression over *LWS2*, we treated embryos with at-RA from 36–75 hpf, and performed *in situs* for *LWS1* and *LWS2*. Interestingly, this RA treatment was by comparison ineffective at inducing *LWS1* expression (Fig 4B). These results support the hypothesis that photoreceptor-generating lineages experience states of shifting plasticity, such that increased RA signaling during the progenitor period influences rod vs. cone fate decisions, while increased signaling during differentiation influences the choice of cone opsin [19]. Alternatively, because fewer LWS cones are generated in embryos treated with RA at 36 hpf [19], effects of RA on *LWS1* may be more difficult to detect after this earlier treatment.

### Sustained RA exposure can cause an LWS2-to-LWS1 “opsin switch” in embryonic LWS cones

We carried out additional experiments in which at-RA or 9-cis RA exposure was continued from 48 hpf to 4 dpf (100 hpf). Control embryos again showed widespread expression of *LWS2*, and essentially no expression of *LWS1* (Fig 3I and 3J). However, some embryos treated with 9-cis RA and examined at 4 dpf displayed a drastic increase in *LWS1* expressing cones, and this appeared to be at the expense of *LWS2* expressing cones (Fig 3K and 3L), suggesting that individual cones had switched expression from the second member of the *LWS* array (*LWS2*), to the first member (*LWS1*). The use of whole mount *in situ* analysis to better quantify numbers of each cone subtype (as in [19,29]) was not possible due to failure of probe penetration in these larger, 4 dpf embryos. Therefore we assigned expression scores to hybridized cryosections. These scores were specific to *LWS1* or *LWS2*, given that in any embryo, *LWS1*, when expressed, was predominantly (though not exclusively) ventral (Fig 3G), and *LWS2*, when expressed, was predominantly dorsal (Fig 3L) (see Materials and Methods for further details). The at-RA or 9-cis RA exposures increased the *LWS1* expression score in nearly all sections examined, and this increase was statistically significant (Fig 4C and Table 6). Treatment with RA correspondingly reduced the *LWS2* expression scores observed 4 dpf, but only for 9-cis RA treatment (Fig 4C and Table 6). Because the effect of 9-cis RA appeared more robust than that of at-RA (Table 6), suggesting the involvement of an RXR, we treated embryos with the selective RXR agonist bexarotene [81] (0.3 μM or 0.06 μM) from 48 hpf to 4 dpf. This agonist significantly upregulated *LWS1* and downregulated *LWS2* (qPCR; p<0.001 vs. DMSO; S1 Fig), consistent with roles for RXRs in regulating the *LWS* array.

We next performed experiments in which the 9-cis RA treatments were continued up to 5 dpf (124 hpf; at-RA treatment for this duration resulted in a high mortality rate). In some of the 9-cis RA-treated embryos, *LWS1* expression appeared to have nearly entirely replaced *LWS2* expression (Figs 3M–3P and 4E, Table 6). Collectively the results of the *LWS* gene-specific *in situ* hybridization studies indicate that exogenous RA is capable of promoting (or de-repressing) expression of *LWS1*, while repressing expression of *LWS2*.

### A GFP reporter for LWS1 is upregulated by RA

We considered that a transgenic tool may offer additional insights into the regulation of the *LWS* opsin gene array by retinoids, and utilized the line, *Tg(LWS1/GFP-LWS2/RFP-PAC(H))#430* (abbreviated hereafter as *LWS*:*PAC(H)*). The genome of this line harbors a ~100kb PAC clone that includes the entire *LWS* array, but with GFP-polyA inserted into the location of the first exon of *LWS1*, and RFP-polyA replacing the first exon of *LWS2* [82]. Expression of GFP and RFP replicate the spatiotemporal expression patterns of *LWS1* and *LWS2*, respectively [82].

**Fig 5:**
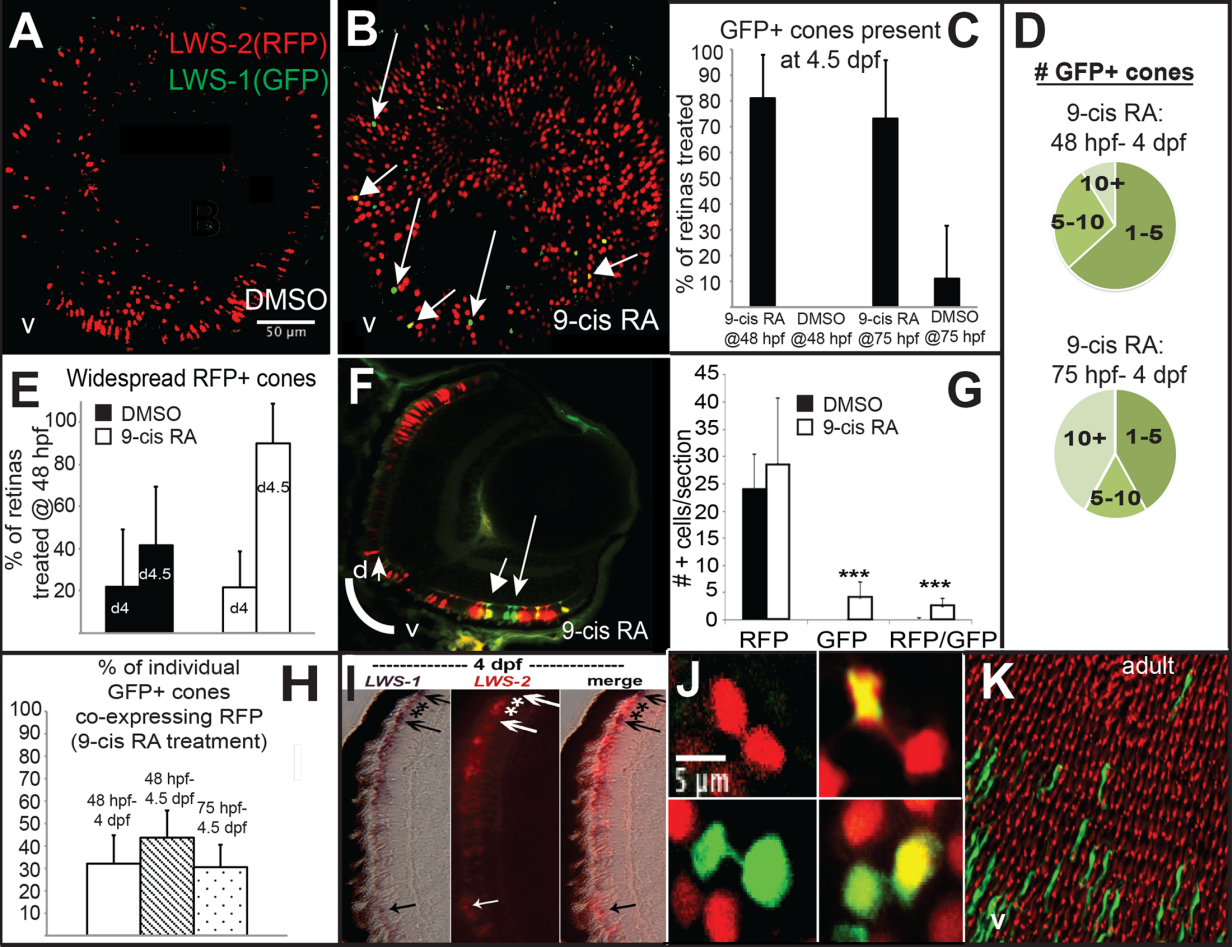
A GFP reporter for *LWS1* indicates a switch from *LWS2* to *LWS1* in response to RA treatment. A-C. Whole mount confocal images of retinas from *LWS*:*PAC(H)* embryos treated with DMSO (A) or 0.3 μM 9-cis RA (B) from 48 to 96 hpf. *LWS2*:RFP+ cones (red) are found in control retinas (A), while cones expressing *LWS1*:GFP alone (green, arrows) or co-expressing *LWS2*:RFP and *LWS1*:GFP (yellow, arrowheads) are found in retinas treated with RA (B); *LWS1*:GFP expression in transgenic retinas treated with RA tend to appear ventrally and peripherally (B). C. Percentage of retinas examined that contain *LWS1*:GFP+ cones for DMSO or 9-cis RA treatment 48 hpf to 4.5 dpf and from 75 hpf to 4.5 dpf. Error bars represent 95% binomial confidence interval. D. The pie charts show the frequency of 9-cis RA treated transgenic retinas expressing the indicated number of *LWS1*:GFP+ cones for treatment 48 hpf to 4.5 dpf (top) or 75 hpf to 4.5 dpf (bottom). E. Graphs show the percentage of *LWS*:*PAC(H)* transgenic retinas that contain widespread (covering more than half of the retina) *LWS2*:RFP+ cones at the indicated time points following treatment beginning at 48 hpf (n = 15, RA 4 dpf; 7, RA 4.5 dpf; 9, DMSO 4 dpf; 4 DMSO 4.5 dpf). Error bars represent 95% binomial confidence interval. F. Indirect immunofluorescence image of a transverse section of an *LWS*:*PAC(H)* embryo treated with 9-cis RA. The arrowhead indicates a cone co-expressing *LWS1*:GFP and *LWS2*:RFP. The arrow indicates a cone expressing *LWS1*:GFP. G. Graph indicating the numbers of *LWS*2:RFP (RFP), *LWS1*:GFP (GFP), or dual label (RFP/GFP) cones per section in retinas treated with DMSO or 9-cis RA (DMSO vs. 9-cis RA ***, p<0.001; 2-tailed Student’s t-test). H. Individual *LWS1*:GFP+ cones from retinas treated with 9-cis RA for the indicated time frames were examined for co-expression of *LWS2*:RFP. I. Dual *in situ* hybridization for *LWS1* and *LWS2* after treatment with 9-cis RA from 48 hpf to 4 dpf. Purple color reaction indicates *LWS1* expression; pink fluorescent color indicates *LWS2* expression; arrows at top show cones expressing *LWS1* only; asterisks (*) show cones expressing *LWS2* only; arrow at bottom shows cone that is dually labeled. J. Confocal images of pairs of cones at the end of mitosis from whole mount transgenic retinas treated with 9-cis RA from 48 hpf to 4.5 dpf. Apparent daughter cells of cone progenitors were observed expressing the same LWS opsin as well as pairs where one daughter cell also co-expresses a different LWS opsin. K. Confocal image of adult *LWS*:*PAC(H)* whole mounted retina showing isolated GFP+ (reporting LWS1) cones.

**Fig 6:**
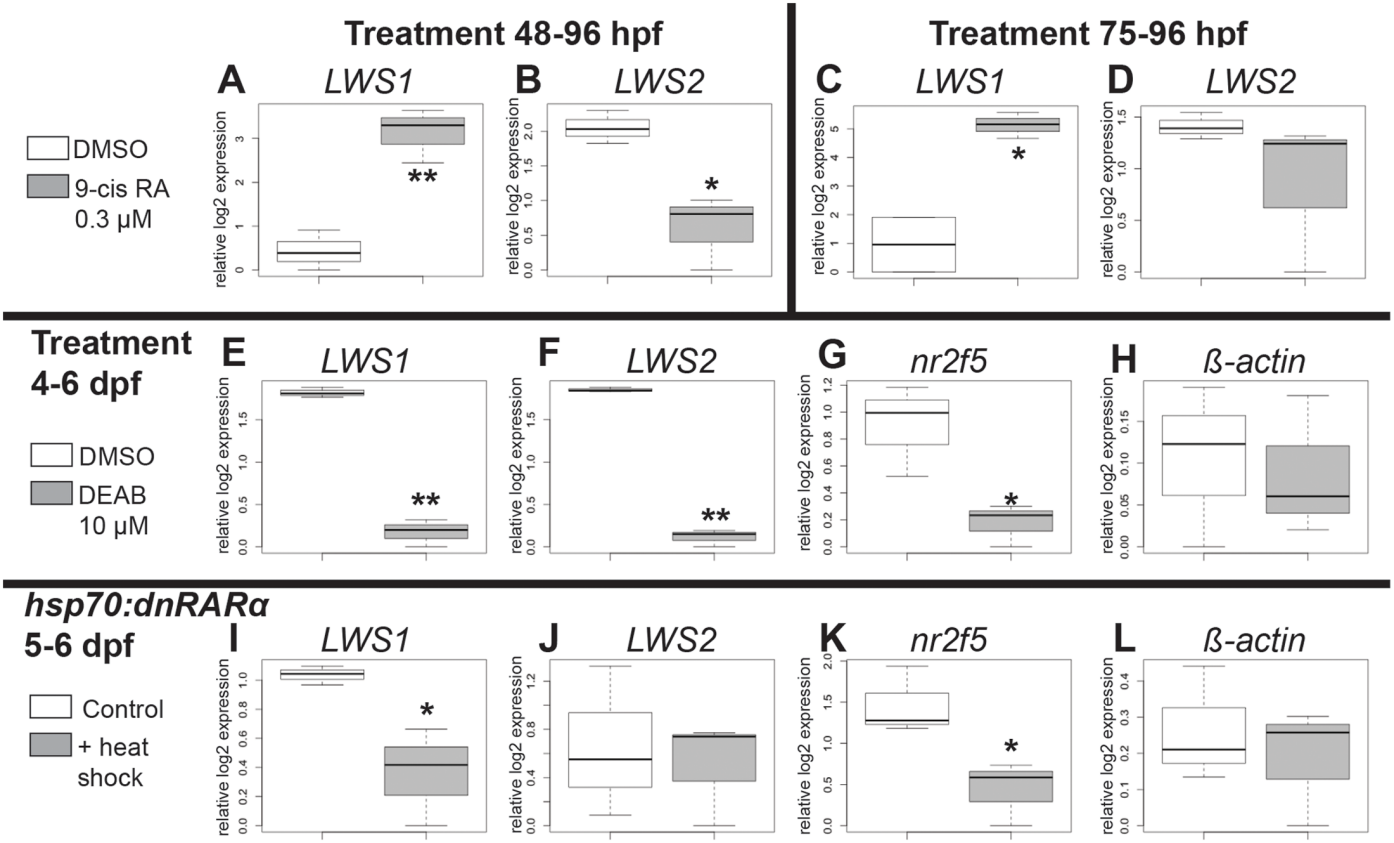
Quantitative PCR for gene expression after retinoid treatment and reduction of RA signaling. Box plots indicate relative log2 expression of the indicated genes. SciH embryos treated with DMSO (white boxes) or 9-cis RA (gray boxes), for 48–96 hpf (A,B) or 75–96 hpf (C,D) were examined for expression of *LWS1* (A,C) and *LWS2* (B,D) following treatment. E-H. SciH embryos were treated with DMSO (white boxes) or DEAB (gray boxes) from 4–6 dpf. Gene expression levels for *LWS1* (E), *LWS2* (F), *nr2f5* (G), and *b-actin* (H) are shown following treatment. I-L. Transgenic *pd5* (*hsp70*:*dnRARα*) embryos were examined for expression levels of *LWS1* (I), *LWS2* (J), *nr2f5* (K), and *b-actin* (L) at 7 dpf after control, no heat shock (white boxes) treatment, or after heat shock on day 5 and day 6 (gray boxes). In the boxplots, the boxes demarcate the 25^th^ and 75^th^ percentiles, dark horizontal lines designate the medians, and whiskers represent the upper and lower limits. *p<0.05, **p<0.01 (2-tailed Student’s t-test).

*LWS*:*PAC(H)* embryos were treated with DMSO or 9-cis RA at 48 hpf and analyzed as whole mounts by confocal microscopy for RFP and GFP fluorescence at 96 hpf (4 dpf). DMSO-treated embryos displayed RFP fluorescence only (reporting *LWS2*; Fig 5A and 5C), while 9-cis RA treated embryos contained some GFP+ (reporting *LWS1*) cones, as well as some doubly-labeled cones (Fig 5B–5D and 5H), often located in ventral (Fig 5B) and peripheral retina (Fig 5B and 5F). In the transgenic *LWS*:*PAC(H)* line, most retinas did not display widespread *LWS2*:RFP cones at 4 dpf regardless of treatment (Fig 5E), in contrast to what was observed for native *LWS2* mRNA (Figs 3 and 4), suggesting that reporter expression may be delayed in onset compared with expression from the endogenous *LWS* array. Even without widespread *LWS2*:RFP expression, retinas from 9-cis RA treated embryos contained *LWS1*:GFP+ cones at 4 dpf (Fig 5B–5D and 5F). In addition, the numbers of RFP-expressing cones were not significantly reduced by 9-cis RA treatment as compared with the numbers in DMSO-treated control embryos (Fig 5G), suggesting that the response of the PAC clone *LWS* transgene array to retinoids may also be slower than that of the native *LWS* array. It is possible that an important regulatory element may be present within the first exons of *LWS1* and/or *LWS2*, and/or that the presence of the GFP or RFP DNA sequences is disruptive. An alternative explanation for the persistence of RFP—when native *LWS2* is downregulated—in RA-treated embryos, is that the RFP protein may be more stable than the *LWS2* mRNA. The fact that nearly half of the GFP+ cones in RA-treated embryos co-express RFP is consistent with this hypothesis (Fig 5H). A time-dependent increase in doubly-labeled *LWS1*:GFP and *LWS2*:RFP cones provides further evidence for a switch from *LWS2* to *LWS1* expression in individual cones upon RA treatment (Fig 5H). Finally, treatment with 9-cis RA beginning at 72 hpf, when embryonic cones are postmitotic [38], resulted in higher numbers of GFP+ cones per *LWS*:*PAC(H)* eye (Fig 5D), and a more robust increase in expression of native *LWS1* mRNA (Fig 6A and 6C), indicating that postmitotic LWS cones may be more competent to express *LWS1* in response to RA treatment. Interestingly, this later (72 hpf) treatment did not result in significant changes to *LWS2* expression (Fig 6B and 6D), similar to the effect of one day of RA exposure, but from 48–72 hpf (Fig 2H and 2K). Either a longer treatment (2 days of exposure) is required for effects of RA on *LWS2*, and/or the plasticity of LWS cones to downregulate *LWS2* in response to RA is developmental stage-dependent.

In order to verify that individual red-sensitive cones switched opsin expression, we performed dual *in situ* hybridizations using 9-cis RA-treated SciH embryos as cryosections at 100 hpf. This approach demonstrated the existence of some cones that were co-labeled with the *LWS1* probe and the *LWS2* probe (Fig 5I), indicating that an individual cone may contain both mRNAs, perhaps during the “switch” triggered by 9-cis RA exposure. During such a switch, the formerly transcribed mRNA may still be present while the newly transcribed mRNA is accumulating [83]. It is also possible that cones expressing both *LWS* genes transcribed *LWS1* from one chromosome, and *LWS2* from the other. In contrast to the situation in humans where the *LWS/MWS* array is X-linked, the *LWS* array of zebrafish is on chromosome 11, an autosome [3].

It was recently demonstrated that embryonic zebrafish *LWS* cones are generated by committed, *TRβ2+* progenitors that undergo a terminal mitotic division to generate a pair of *LWS* cones that maintain a narrow cytoplasmic attachment as they begin to differentiate [17]. To further address whether the decision to express *LWS1* or *LWS2* takes place in the progenitor cells or in the postmitotic, differentiating cones, we visually inspected confocal images of eyes derived from RA-treated *LWS*:*PAC(H)* embryos for the presence of pairs of cones showing this cytoplasmic attachment (Fig 5J). We observed pairs of daughter cells in each of the following combinations: 1) both expressing *LWS2*:RFP; 2) both expressing *LWS1*:GFP; 3) pairs in which one daughter cell expresses both *LWS2*:RFP and *LWS1*:GFP while the other cell expresses only *LWS2*:RFP or only *LWS1*:GFP (Fig 5J). These results suggest that the decision of *LWS1* vs. *LWS2* can likely be altered post-mitotically in differentiating cones by RA treatment, although this finding does not rule out effects on cone precursors as well. In support of post-mitotic regulation of *LWS* expression, adult *LWS*:*PAC(H)* (untreated) whole retinas showed many examples of GFP+ cones that were surrounded by RFP+ cones (Fig 5K), consistent with the daughter cells of an *LWS* cone progenitor adopting asymmetric fates, or with switches in *LWS* gene expression taking place in fully differentiated cones later during animal growth.

### RA-induced LWS1-expressing cones do not disrupt the LWS cone mosaic

**Fig 7:**
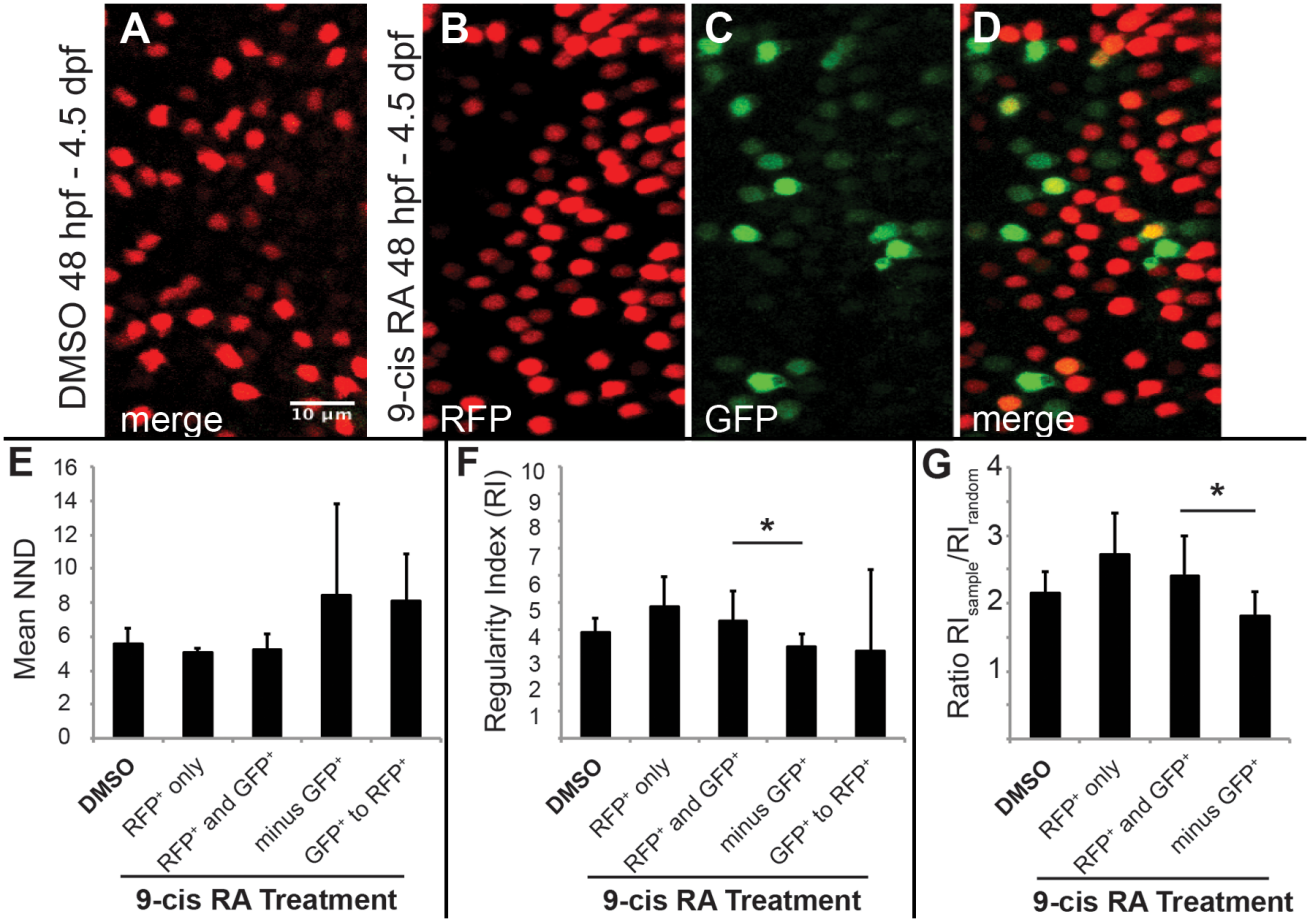
Two-dimensional pattern analysis of LWS cones in *LWS*:*PAC(H)* embryos exposed to RA: Retinoic acid induced LWS1-expressing cones do not disrupt the LWS2 cone mosaic. Regions from confocal images at 60X magnification obtained from whole mounted *LWS*:*PAC(H)* eyes treated with DMSO or 0.3 μM 9-cis RA 48 hpf to 4.5 dpf were used for pattern analysis. A-D. Representative regions used to determine the mean nearest neighbor distance (NND) and Regularity Index (RI) of LWS cones in control and 9-cis RA-treated eyes. Regions from DMSO treated retinas expressed only *LWS2*:RFP cones (red) (A), while regions from 9-cis RA treated retinas (B-D) contained RFP+ (red), GFP+ (green), and RFP+/GFP+ (yellow) cones. B-D. Images showing RFP signal only (B), GFP signal only (C), and merge of both signals (D) in regions of 9-cis RA treated retina. Graphs of NND (E, left three bars) and RI (F, left three bars) corresponding to regions from DMSO treated (DMSO) and 9-cis RA treated retinas. 9-cis RA treated retinas were further divided into groups: regions of retina where only RFP+ cones are present (RFP+ only), and regions containing both RFP+ and GFP+ cones (RFP+ and GFP+) when all labeled cells (regardless of RFP or GFP expression) are treated as the same cell type. Graphs also indicate NND (E, right two bars) and RI (F, right two bars) for 9-cis RA treated retinas when GFP+ cones are subtracted from the RFP+ and GFP+ mosaic (minus GFP+) and when the pattern of GFP+ cones to RFP+ cones (GFP+ to RFP+) is analyzed. G. Graph showing the ratio of RI from each indicated region (RI_sample_) with that of the average RI from 1000 generated random samples of the same number of cells (RI_random_). The asterisk (*) in F and G indicates p<0.05 for a two-tailed Student’s t-test between the indicated groups. Error bars represent standard deviation (n = 7 regions from 4 eyes, DMSO; 6 regions of RFP+ only and 10 regions RFP+ and GFP+ from 6 eyes, RA).

The presence of cones co-expressing *LWS1* and *LWS2* (Fig 5I), or GFP and RFP in the *LWS*:*PAC(H)* line (Fig 5B and 5F) following RA treatment suggests that RA promotes an opsin switch in LWS cones rather than *LWS1* expression in other cone subtypes. We therefore hypothesized that RA treatment would not disrupt the patterns of LWS cones. We suspected this was the case based upon our prior study showing that the two-dimensional spatial arrangement of LWS cones was not different in embryos treated with RA at 48 hpf, as compared with controls [29]. In order to perform spatial pattern analysis with a sufficient number of GFP+ cones, we treated *LWS*:*PAC(H)* embryos with DMSO or 9-cis RA from 48 hpf to 4.5 dpf and imaged whole eyes by confocal microscopy. At 4.5 dpf, many retinas from embryos treated with 9-cis RA contained regions with 10 or more GFP+ cones (Fig 7B–7D), while retinas from DMSO treated embryos only contained RFP+ cones (Fig 7A). The developing RPE and iridophores at this later sampling time made it impossible to analyze whole eyes, so instead we obtained high resolution images at 60X magnification using regions clear of RPE and iridophores for spatial analysis (Fig 7A–7D). For this analysis, the “GFP+” category included cones that express GFP only or those that express both GFP and RFP.

Average nearest neighbor distances (NNDs) were similar for patterns found in retinas from DMSO treated and 9-cis RA treated embryos even when GFP+ cones were included in the analysis and treated as the same cell type as RFP+ cones (Fig 7E), suggesting that the GFP+ cones did not disrupt the LWS cone pattern and therefore constitute the same cone population. To further test this hypothesis we calculated regularity indices (RIs); the RI is the mean NND divided by the standard deviation, adjusted for object density. To ensure that RIs of selected regions could indicate patterns of regularity, each region selected for analysis was compared against 1000 random simulations of the same number of objects (Fig 7G). The RIs were similar for patterns found in retinas from DMSO treated and 9-cis RA treated embryos even when GFP+ cells were included in the spatial analysis and treated as the same cell type as RFP+ cones (Fig 7F and 7G). Further, when the GFP+ cones were removed from the combined GFP+ and RFP+ mosaic of 9-cis RA treated retinas, the pattern was disrupted as indicated by an increase in average NND and its standard deviation (Fig 7E) along with a significant reduction in the RI (Fig 7F and 7G). These findings indicate that *LWS1* expressing cones observed in RA-treated retinas are in the same cone population as *LWS2* cones, and that non-*LWS* photoreceptor types are not recruited by RA treatment to express *LWS1*.

We also performed cross-correlative NND analysis of GFP+ cones in relation to RFP+ cones in 9-cis RA treated retinas (Fig 7E and 7F; “GFP+ to RFP+”). The cross-correlative NNDs and RIs were not significantly different from the auto-correlative metrics, again suggesting that the GFP+ cones are in the same cone population as the RFP+ cones.

### Reduced RA signaling prevents upregulation of LWS1 during larval development

To investigate endogenous roles for RA signaling in the regulation of the tandemly-duplicated *LWS* opsin genes, we treated zebrafish with the RA synthesis inhibitor, DEAB [84] (10 μM), from 4–6 dpf, the time of initial expression of *LWS1* [12]. DEAB-treated larvae showed significantly reduced expression of both *LWS1* and *LWS2* mRNA (Fig 6E and 6F), suggesting that endogenous RA signaling is involved in regulating expression from the *LWS* array. To confirm that DEAB treatment reduced RA signaling, we verified that this treatment also resulted in significantly reduced expression of the RA signaling target *nr2f5* mRNA ([77]; present study) as compared to levels in controls (Fig 6G). An additional control gene, *β-actin*, was not affected by the DEAB treatment (Fig 6H).

As a complementary loss-of-function strategy, we used the transgenic line *pd5*, which harbors a heat shock-driven dominant-negative (human) *RARα* transgene [85]. Heat-shocked *pd5* larvae also showed significantly reduced expression of *nr2f5* as compared to non heat-shocked *pd5* controls, providing evidence that a known target of RA signaling was affected by the dominant-negative effect of an overexpressed truncated RAR (Fig 6K). Expression of β-actin was not affected by heat-shock (Fig 6L). The heat-shocked larvae showed significantly reduced expression of *LWS1* as compared to controls (Fig 6I), suggesting that an endogenous retinoid receptor [or other nuclear hormone receptor(s)] is/are required for activation of *LWS1* at the onset of its expression. Interestingly, expression of *LWS2* mRNA was not affected in heat-shocked *pd5* larvae (Fig 6J). Together these results demonstrate an endogenous role for RA signaling in upregulating *LWS1* at the onset of its expression, but suggest additional factors may participate in the ongoing regulation of *LWS2* expression.

### Expanded RA signaling domain in RA-treated embryos matches the expanded LWS1 expression domain

**Fig 8:**
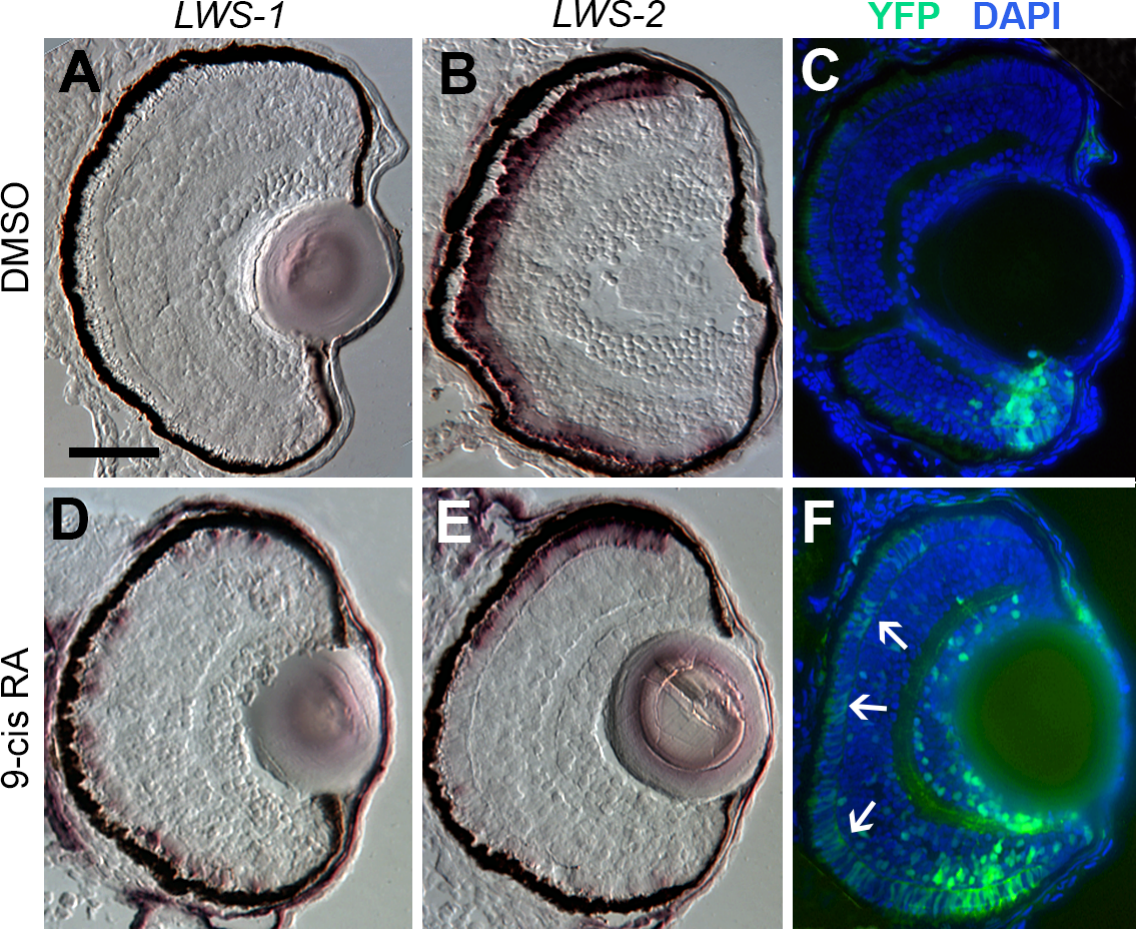
Spatial relationship of RA signaling activity compared with expression of *LWS1* and *LWS2*. A-C. Sections obtained from a RARE:YFP transgenic embryo treated with DMSO 48 hpf-100 hpf, then labeled for *LWS1* mRNA expression (A), *LWS2* mRNA expression (B), or YFP immunofluorescence (C; counterstained with DAPI). D-F. Sections obtained from a *RARE*:*YFP* transgenic embryo treated with 0.3 μM 9-cis RA 48 hpf– 100 hpf, then labeled for *LWS1* mRNA expression (D), *LWS2* mRNA expression (E), or YFP immunofluorescence (F). Expanded RA signaling domain (arrows) is similar to the *LWS1* expression domain. Scale bar in A (applies to all) = 50 μm.

The transgenic zebrafish line, *RARE*:*YFP*, permits the visualization of active RA signaling within individual tissues and cells, and has previously been used to demonstrate native RA signaling domains predominantly in ventral, and less evidently in dorsal retina during zebrafish development [19,29,33,84]. It is interesting that these native RA signaling domains anticipate the native expression domains of *LWS1* [12], consistent with potential endogenous regulatory mechanisms. We treated *RARE*:*YFP* embryos with at-RA, 9-cis RA, or DMSO at 48 hpf, and fixed embryos for cryosectioning at 100 hpf. In control embryos, *LWS1* expression had not yet commenced, and *LWS2* expression was found throughout the ONL (Fig 8A and 8B), similar to the situation for wild-type embryos (compare to Fig 3I and 3J). The RA signaling domain, as revealed by YFP fluorescence, was confined to ventral retina (Fig 8C). As shown previously [19,29], this signaling domain included some cells in the ONL. In RA-treated embryos, *LWS1* expression was extensive, although not found throughout the entire ONL, while *LWS2* expression was more restricted (Fig 8D and 8E), similar to the response of wild-type zebrafish (compare to Fig 3K and 3L). The YFP+ RA signaling domain was greatly expanded, particularly in the ONL (Fig 8F), and appeared to roughly match the expanded *LWS1* expression domain (Fig 8D). Therefore, cells located in the ONL both increase RA signaling and express *LWS1* following RA treatment.

### Endogenous RA signaling domain of juvenile zebrafish retina coincides with zone of native LWS1 expression

**Fig 9:**
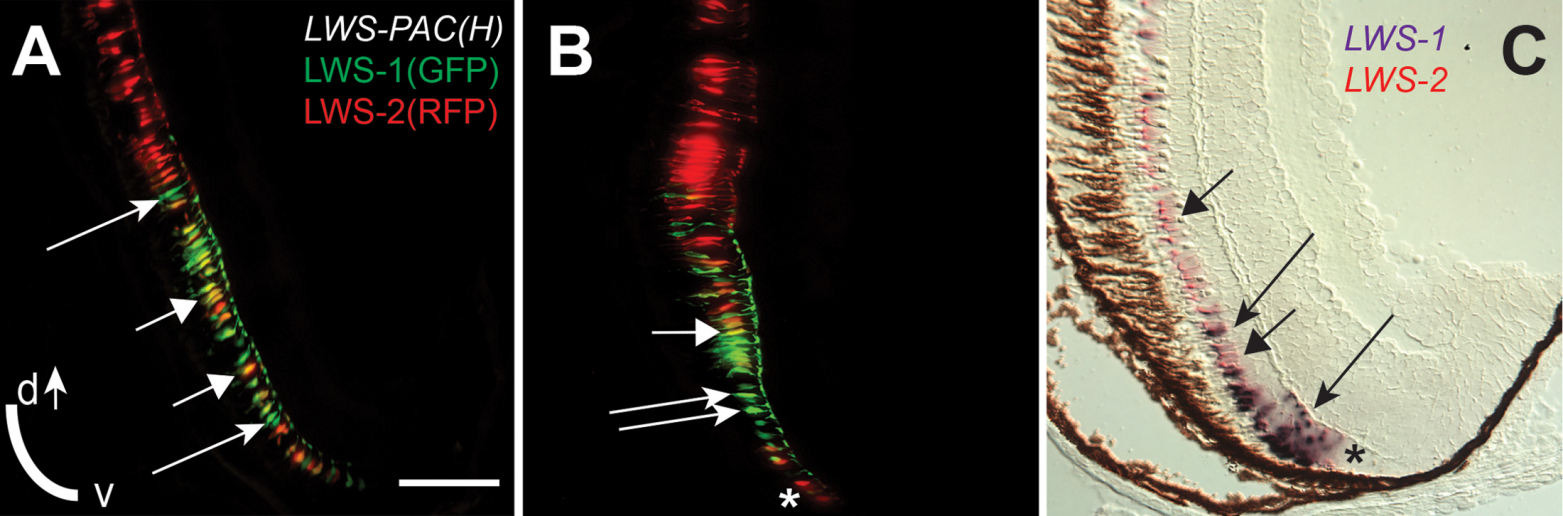
An “LWS Transition Zone” exists in ventral retina of juvenile fish. Sections of retina from one-month old juvenile retinas are shown. A and B. Two different sections from separate *LWS*:*PAC(H)* transgenic fish showing *LWS1*:GFP+ cones (green fluorescence; long arrows), *LWS2*:RFP+ cones (red fluorescence), and cones co-expressing *LWS1*:GFP and *LWS2*:RFP (yellow, short arrows). B. The asterisk (*) indicates a cone expressing only *LWS2*:RFP at the ventral periphery. C. Dual *in situ* hybridization using cDNA probes for *LWS1* (purple) and *LWS2* (red/pink). Cones expressing *LWS1* only are indicated by long arrows; cones co-labeled with both probes are indicted by short arrows. The asterisk (*) indicates an *LWS2* singly labelled cone at the ventral periphery. Scale bar in A (applies to all) = 25 μm; v, ventral; d, dorsal.

During larval and juvenile growth of the zebrafish retina, the relative size and shape of the domain of *LWS1* expression in red-sensitive cones enlarges to include the ventral ¼ of the retina at the dorsal-ventral midline [12]. The expression of the *LWS* genes during retinal growth must therefore be dynamic, as the circumferential germinal zone (CGZ) adds new neurons (including cones) at the periphery [10]. We verified that the *LWS1* expression domain could be visualized in juvenile (one month old) *LWS*:*PAC(H)* transgenics, and discovered an “*LWS* transition zone” in ventral retina in which many LWS cones co-expressed *LWS1* and *LWS2* (Fig 9A–9C). This finding is consistent with opsin switching in postmitotic cones as a mechanism for altering the relative sizes of the domains of expression of each *LWS* gene during retinal growth. In some cryosections, single RFP+ (reporting *LWS2*) cones were observed at the farthest ventral periphery, adjacent to GFP+ (reporting *LWS1*) cones (Fig 9B). Dual *in situs* for the endogenous *LWS1* and *LWS2* mRNAs confirmed that the most recently-generated LWS cone—located closest to the CGZ—could be an *LWS2*+ cone (Fig 9C). Therefore, *LWS2* expression may constitute the initial or default opsin expression status of newly-generated LWS cones, which in ventral retina then switch to express *LWS1* as the retina grows and new cones are added.

**Fig 10:**
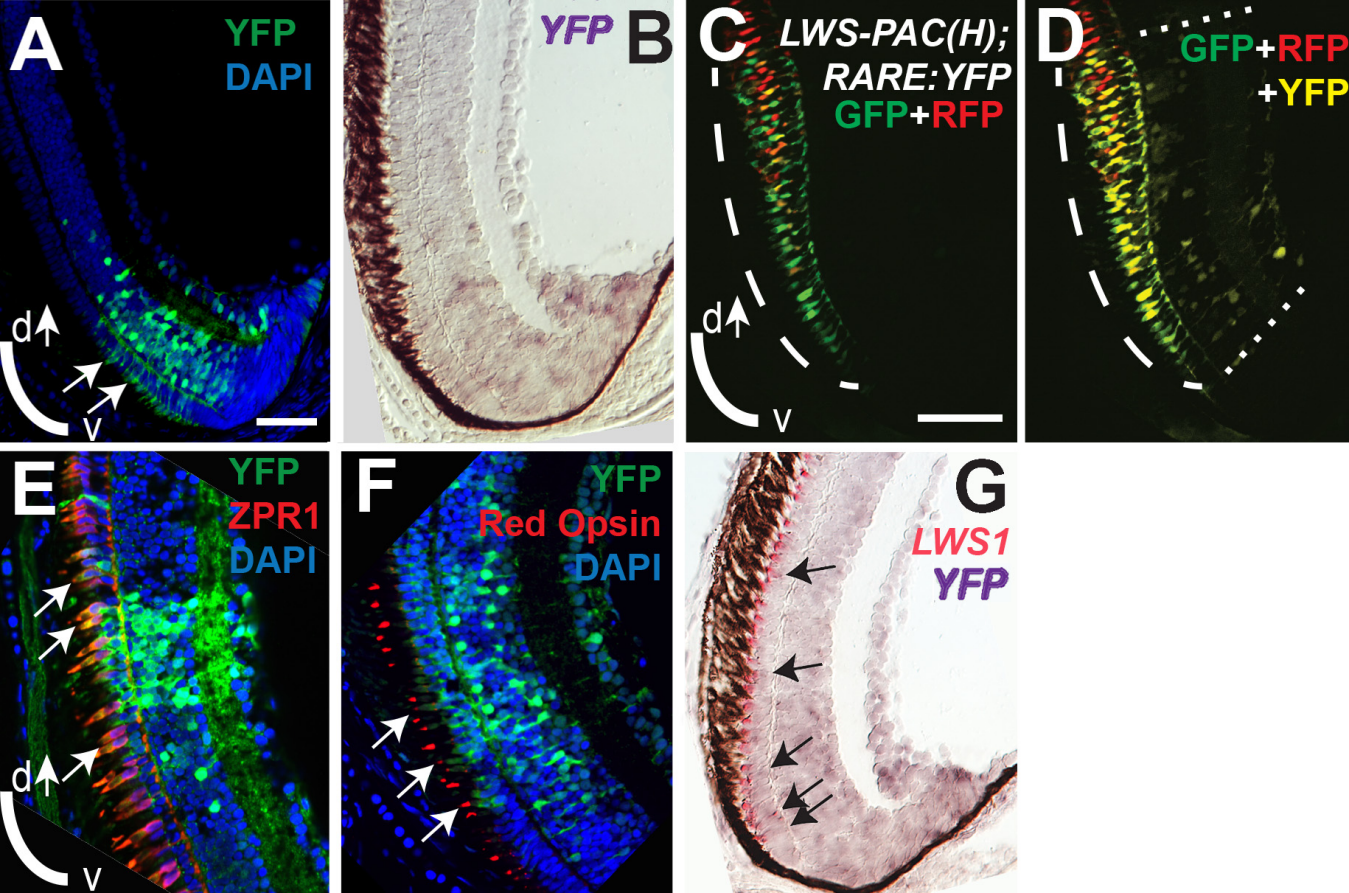
RA signaling continues in juvenile retinas and the signaling domain includes red opsin+, *LWS1*+ cones. Sections of retina from one-month old fish are shown, with ventral pole at bottom of images. A. Immunocytochemistry to reveal YFP signal and DAPI in a section from *RARE*:*YFP* retina. Arrows indicate YFP signal in the outer nuclear layer, corresponding to the location of cone photoreceptors. B. *In situ* hybridization for *topaz-YFP* mRNA in a section from a *RARE*:*YFP* retina. C and D. *LWS*:*PAC(H)* were crossed with *RARE*:*YFP* and doubly transgenic juvenile retina sections were examined for RFP, GFP, and YFP fluorescence. Although YFP and GFP cannot be fully resolved, the YFP+ signaling domain coincides with the GFP (LWS1) expression domain. The GFP expression domain (reporting LWS1) is designated by dashed arc (C and D), while the YFP domain (reporting RA signaling) is between the dashed straight lines (D). E. Sections from *RARE*:*YFP* juvenile retinas were stained with antibodies for YFP, ZPR1 (labels red- and green-sensitive cones), and DAPI; merged image is shown. Colabel of YFP and ZPR1 is yellow. Arrows indicate ZPR1+ cones that are YFP+. F. Sections from *RARE*:*YFP* juvenile retinas were stained with antibodies for YFP, red opsin (pan-LWS), and DAPI; merged image is shown. Arrows indicate red opsin+ cone outer segments that are continuous with YFP+ cell bodies. G. Dual *in situ* hybridization for *topaz-YFP* (purple) and *LWS1* (red/pink) mRNA in a section from one-month old *RARE*:*YFP* retina. Arrows indicate examples of some of the colabeled cones. Scale bar in A = 25 μm; scale bar in C (applies to B-G) = 25 μm; v, ventral; d, dorsal.

To evaluate endogenous roles for RA signaling in dynamic regulation of the *LWS* opsin genes during retinal growth, we used the *RARE*:*YFP* transgenic line to localize RA signaling domains in one month old zebrafish. In three fish sampled as cryosections, a YFP+ retinal region was detected in a radial strip of ventral retina, located near and sometimes including the CGZ (Fig 10A). The location of this RA signaling domain was confirmed by *in situ* hybridization for the mRNA encoding the *YFP* reporter (Fig 10B). The spatial relationship of the RA signaling domain, as compared to that of the *LWS1* expression, was determined in one month old *RARE*:*YFP; LWS*:*PAC(H)* zebrafish. In these fish, YFP fluorescence coincided with the most peripheral region of *LWS1* expression (reported by GFP) (Fig 10C and 10D), consistent with endogenous roles for retinal RA signaling regulating differential expression of the *LWS* opsin genes during retinal growth.

We wished to determine whether the cones engaged in RA signaling (YFP+) corresponded with those expressing *LWS1* (GFP), but the YFP and GFP fluorescent signals were challenging to unambiguously resolve (Fig 10C), even on a spectral scanning confocal microscope. As alternative strategies, we first confirmed that some of the YFP+ cells of the ONL were LWS cones by colabeling experiments in one month old *RARE*:*YFP* zebrafish, detecting YFP with indirect immunofluorescence, along with indirect immunofluorescence labeling for the double cone marker ZPR1 [86] (Fig 10E) or a pan-LWS antibody [80,82] (Fig 10F, “Red Opsin”). Next we performed dual *in situs* for the *YFP* mRNA together with *LWS1* mRNA, and could detect colabeled cells in the ONL (Fig 10G). Collectively the evaluation of juvenile zebrafish retinas supports endogenous functions for RA signaling in regulating differential expression of the tandemly-duplicated *LWS* opsin genes.

## Discussion

Using microarray analysis, this study identified several eye-specific regulatory target genes for RA signaling during the period of embryonic photoreceptor differentiation in the zebrafish. Among these targets was *opn1lw1/LWS1*, the first member of the tandemly-duplicated genes encoding the long wavelength-sensitive opsin proteins. The results of subsequent qPCR, *in situ* hybridization, dual *in situ* hybridization, and the use of selected transgenic lines that report *LWS1* vs. *LWS2* expression and RA signaling, collectively indicated that increased RA signaling was associated with increased expression of *LWS1* while decreasing expression of *LWS2* in individual LWS cones. Loss-of-function approaches confirmed that endogenous RA signaling was required for promoting the onset of *LWS1* expression. The coincidence of an endogenous RA signaling domain with the domain of *LWS1* expression is consistent with a role for RA signaling in regulating ontogenetic changes in *LWS* opsin expression during normal animal growth. To our knowledge this is the first report of a developmental cell signaling system regulating differential expression of tandemly-duplicated opsin genes.

### Ocular targets of RA signaling in the zebrafish embryo

Our microarray analysis identified multiple targets of exogenous RA that encode enzymes and transcription factors involved in regulating RA concentrations and RA signaling, including *aldh1a2*, *cyp26a* and *b*, *dhrs3a*, and *rxrγa* (Table 2). In most cases the transcriptional response suggested a metabolic attempt to restore RA homeostasis, and several of these genes were also differentially expressed in response to RA in embryos undergoing somitogenesis [78] and in larval zebrafish heart [77]. These data demonstrate the existence of homeostatic mechanisms to maintain RA levels and/or gradients, and that these mechanisms are conserved within the eye and elsewhere.

The strategy of using whole dissected eyes as starting material for the microarray resulted in the detection of several RA signaling targets that are ocular but likely extraretinal. These targets include genes involved in migration and differentiation of the neural crest-derived periocular mesenchyme and its derivatives [46,47], already known to be regulated by RA. Genes expressed in vascular and hematopoietic lineages (*epo*, *angptl5*) are known to be regulated by RA in other organs [87,88], and lens crystallins (though not the specific crystalline genes detected in the present microarray) have been demonstrated as targets of RA signaling [89].

Components of several cell signaling systems were differentially expressed in eyes of RA-treated embryos, most notably two *bmps* (*bmp2b*, *bmp4*), extracellular modifiers of Wnt signaling, and *dio2*, encoding a deiodinase enzyme that locally converts thyroxine (T4) to tri-iodothyronine (T3; thyroid hormone). All four of these signaling pathways—RA, Bmps, Wnts, and T3 –play roles in dorsal-ventral patterning of the optic vesicle and/or retina [16,24,67,90]. Changes in expression of *bmps*, and of modifiers of Wnt and T3 signaling, in response to exogenous RA suggest mechanisms for coordination of these signaling pathways in the eye during photoreceptor differentiation, as well as the potential for interactions among these signaling pathways.

A final interesting set of RA signaling target genes within the zebrafish eye were those encoding retinal transcription factors. For example, genes encoding three nuclear hormone receptors (*nr0b2a*, *nr2f5*, *nrip1b*) [56] were upregulated, along with the eye field transcription factor *six6a* [51,91]. *Rxrγa* was upregulated by the RA treatment (and is expressed transiently in the ONL of zebrafish embryos [19]). Interestingly, RXRγ in mouse retina is required to prevent expression of S- (blue) opsin in mouse cones [6]. It is possible that the observed decrease in differentiation of SWS1 (UV) and SWS2 (blue) cones in zebrafish in response to RA treatment [29] is mediated by RXRγa. Alternatively, or in addition, RXRγa may be involved in mediating the effects of retinoid signaling on expression of *LWS1* and *LWS2*.

### RA signaling regulates differential expression of the tandemly duplicated LWS genes

Excluding genes encoding RA metabolizing enzymes and *hox* genes, the “top hit” of the microarray analysis was *opn1lw1*, or *LWS1* (S1 Table). *LWS1* is the first member of the tandemly-duplicated *LWS* cone opsin array and is normally not expressed until larval stages. In the adult zebrafish eye *LWS1* is restricted to ventral and peripheral retina [3,12]. In the present study, however, treatment with 9-cis RA over a 2–3 day period of embryonic photoreceptor differentiation resulted in significant changeover from *LWS2* to *LWS1* expression, suggesting that RA signaling may act as a molecular regulator of the *LWS* array. The greatly enlarged *LWS1* expression domain in some embryos—occasionally spanning the entire retina—exceeded the size of the *LWS1* expression domain ever observed in native retina [12], indicating that the effect of RA was not simply an acceleration of the normal developmental program. Dual *in situ* hybridization experiments and confocal analysis of *LWS* reporter expression in the *LWS*:*PAC(H)* transgenic line indicated that this switch takes place within individual, postmitotic cones of a single cone population.

Reduction of RA signaling, achieved using two complementary loss-of-function strategies, consistently reduced *LWS1* expression at the time of its normal onset, supporting endogenous functions for RA signaling in promoting *LWS1* expression. RA signaling domains in untreated embryos, RA-treated embryos, and in the growing juvenile retina, which predict or coincide with *LWS1* expression, provided further evidence for such endogenous functions. However, the RA signaling loss-of-function experiments reported here do not demonstrate opposite effects on *LWS2* expression as compared to RA signaling gain-of-function. Reduced RA synthesis (DEAB treatment) downregulated *LWS2* as well as *LWS1*, and overexpression of a dominant-negative RARα caused no significant changes in *LWS2* expression. It is possible that *LWS2* expression is regulated by additional factors or by a different complement of nuclear hormone receptors than is *LWS1*, or that our assays were not timed to capture effects of reduced RA signaling on *LWS2*. The requirement for sustained RA exposure time in embryos (2 days but not 1 day) to decrease *LWS2* supports this interpretation. It is also possible that unliganded RA receptors act to repress the entire array, while liganded RA receptors preferentially activate *LWS1* over *LWS2*. Identification of the receptor(s) mediating these effects will be required to pursue this latter hypothesis.

In the zebrafish retina, increased RA signaling therefore has roles in 1) promoting the rod fate at the expense of cone fates [19], 2) promoting differentiation of rods while slowing the differentiation of blue and UV cones [29,30], and 3) favoring the expression of *LWS1* over that of *LWS2* in individual red-sensitive cones, although not promoting *LWS* opsin expression in other photoreceptor types (present study). It is possible that during evolution of the vertebrate eye, co-option of endogenous RA signaling emerged as a versatile mechanism for regulating multiple sequential patterning events over developmental time. In support of this idea, the spatial pattern of the endogenous RA signaling domain in embryos matches that of the initial pattern of rods [19,79], and anticipates the pattern of *LWS1* expression [12]. The tandemly quadruplicated *RH2* (green-sensitive) opsin genes of the zebrafish also show concentric expression domains with a dorsal-ventral gradient [12], but it is not known whether this array responds to RA signaling.

The present study informs our understanding of the transcriptional network underlying photoreceptor fate determination [13], by suggesting a novel mechanism for choice of tandemly replicated opsin (in humans, *LWS* vs. *MWS*). The current model for *LWS* vs. *MWS* opsin gene regulation in humans assumes that during cone differentiation, a stochastic event favors an association of the upstream locus control region (LCR) with the *LWS* or most proximal *MWS* opsin gene, and that this association becomes permanent [20,92,93]. In humans, the LWS:MWS ratio >1.0 suggests a simple proximity argument: the LCR is closer to the *LWS* gene and therefore more likely to interact with it and promote transcription. However, there is evidence that this stochastic model may not be entirely accurate. For example, the LWS:MWS ratio varies as a function of retinal eccentricity, with highest LWS:MWS ratios in the periphery [9]. The LWS:MWS ratio also varies as a function of ethnic descent [94], a variability not explained by any differences in *LWS* vs. *MWS* gene order, distance from LCR, or promoter sequence [94,95]. These data suggest that a nonrandom *trans* regulatory mechanism may instead regulate *LWS* vs. *MWS* opsin expression. Here we have identified a potential mechanism in the zebrafish, in which RA signaling regulates expression of the orthologous locus containing *LWS1* and *LWS2*. The *LWS* array of zebrafish contains numerous consensus retinoic acid response elements (RAREs; S2 Fig), suggesting the potential for direct regulation of each array by RA in conjunction with an appropriate receptor homo- or heterodimer. However, direct interactions of any RAR or RXR with an opsin gene regulatory region have not been demonstrated, and so this idea remains highly speculative.

The observation that pharmacological agents such as retinoids can control photoreceptor fates [19], differentiation [27–30], and now choice of tandemly replicated opsin (the present study) suggests the potential for pharmacological manipulation of photoreceptor phenotypes in conjunction with anticipated regenerative therapies for human retinal disease [96]. Because nuclear hormone receptors in general respond to pharmacological treatments, they are considered attractive candidates for manipulating photoreceptor phenotype in the treatment of retinal disease [97]. These findings have already seen applications in the generation and differentiation of photoreceptors from human embryonic stem (ES) cells and induced pluripotent stem (iPS) cells [34]. Regulation of human *LWS/MWS* expression in concert with regenerative therapies will be important for the re-establishment of high acuity trichromatic color vision. In addition there is potential for correction of color vision anomalies or X-linked cone dystrophy in individuals with defects in one or two genes of the *LWS/MWS* array (that could be pharmacologically suppressed), but also with one or two normal genes (that could be pharmacologically activated) [98,99], as an alternative to gene therapy [100].

## Materials and Methods

### Animals

Zebrafish were maintained in monitored aquatic housing units on recirculating system water at 28.5°C. Embryos were collected according to Westerfield [101], with light onset considered to be zero hours postfertilization (hpf) and embryonic age timed accordingly thereafter. Embryos used for whole mount analyses were kept transparent by incubating them in system water containing 0.003% phenothiourea (PTU) to inhibit melanin synthesis [101]. All experiments using animals were approved by the University of Idaho’s Animal Care and Use Committee.

Wild-type embryos were of an in-house outbred strain originally obtained from Scientific Hatcheries (now Aquatica Tropicals, Plant City, FL) and are referred to as SciH. The transgenic zebrafish line *RGnY* was generously provided by Elwood Linney. The transgene consists of three copies of retinoic acid response elements (RAREs) derived from the mouse RARβ gene, a zebrafish basal promoter, a Topaz YFP sequence, an SV40 polyadenylation signal, and a small t intron [84]. The endogenous expression patterns of YFP in these fish are consistent with known areas undergoing RA signaling [29,84] and YFP reporter expression increases in response to exogenous RA [19,84]. We refer to this line as *RARE*:*YFP*. In the transgenic line *Tg(LWS1/GFP-LWS2/RFP-PAC(H))#430*, the “transgene” consists of a PAC clone in which the first exons of *LWS1* and *LWS2* were replaced with GFP and RFP, respectively, each followed by a polyadenylation sequence [82]. The spatiotemporal expression patterns of GFP and RFP replicate endogenous patterns of *LWS1* and *LWS2* [82]. We refer to this line as *LWS*:*PAC(H)*. The transgenic zebrafish line *Tg(hsp70*:*dnRARα*, *cryaa*:*GFP)pd5* [85] was obtained from the Zebrafish International Resource Center (ZIRC), and we refer to it as *pd5*. The transgene consists of a heat shock (*hsp70*) promoter driving expression of a dominant-negative (truncated) human RARα, and a separate cassette containing *cryaa* promoter driving GFP constitutively in the lens [85,102]. When *pd5* transgenics are subjected to heat shock, expression of *dnRARα* causes global deficiency in RA signaling [85].

### Retinoid and DEAB treatments and heat shock

Stock solutions of all-trans retinoic acid (at-RA), 9-cis retinoic acid (9-cis RA), bexarotene, and diethylaminobenzaldehyde (DEAB; Sigma, St. Louis, MO) were prepared in dimethylsulfoxide (DMSO; Sigma) and stored under nitrogen in the dark at -20°C. Prior to treatment, embryos were manually dechorionated, and then stock solution was added to the water to result in the final concentrations indicated in Results (DMSO was at a final concentration of 0.1%). The 0.3 μM concentration of RA used in the microarray experiment influences photoreceptor fate and differentiation in zebrafish embryos [19,29,30,33]. For treatments lasting longer than one day, solutions were refreshed every 24 hrs.

Heat shocks (to induce expression of dnRARα in the *pd5* transgenics and result in RA signaling loss-of-function) were performed at 5 and 6 dpf by transferring embryos to 37°C for 1 hr.

### Microarray and analysis

Three sets of parents from the SciH line were used to obtain three clutches of embryos. Each clutch was separated into control and experimental groups, which were treated from 48 to 75 hpf with DMSO (control) or 0.3 μM at-RA (experimental). This generated three biological replicates of control and experimental treatments for six total treatment groups. At 75 hpf, embryos were placed in scintillation vials and snap-frozen by immersing the vial into liquid nitrogen. The vial was filled with methanol pre-chilled on dry ice and embryos were stored at -80°C. After seven days, eyes were dissected from embryos using fine forceps [103,104]. Each treatment group consisted of 64 eyes. Eyes were homogenized using a roto-homogenizer and total RNA was extracted using the RNeasy Kit (Qiagen, Valencia, CA) and quantified (Nano-Drop 1000; Thermo Scientific, Wilmington, DE).

A total of 80 ng of RNA from each treatment group was analyzed to verify quality (Agilent Bioanalyzer, Agilent Technologies, Inc., Wilmington, DE), and amplified using the NuGen kit (San Carlos, CA). Gene expression in the six eye-specific samples was determined using Affymetrix GeneChip Zebrafish Genome Arrays (Affymetrix, Santa Clara, CA). The amplification and microarray procedures were performed at the Genomics Core of the Center for Reproductive Biology (Washington State University, Pullman, WA). The raw data were adjusted for background, normalized, and visualized as RMA (Robust Multi-array Average) using the Bioconductor package in R [105]. To identify differentially expressed genes we used significance analysis of microarray (SAM; [106]). The results were analyzed at False Discovery Rates (FDR) of 10% and 20%. Probe data were annotated using the Zebrafish Genome Assembly version Zv8 (Sanger Institute). The microarray data are available in the NCBI Gene Expression Omnibus (GEO; accession #GSE63873). A gene ontology (GO) analysis was performed using GOEAST (Gene Ontology Enrichment Analysis Software Toolkit), an online tool [39], with a focus on biological processes enriched in the set of differentially expressed genes.

The data were compared to those obtained using Affymetrix platform-based microarray analysis of RA treatments in zebrafish embryos, over different developmental times or in different tissues [77,78]. Raw data were accessed from GEO, were adjusted for background, normalized, and visualized as RMA expression values, and differentially expressed genes were identified using SAM [106]. Genes that were differentially expressed at 10% FDR in two or more datasets ([77,78] and current dataset) were considered potential common regulatory targets of RA signaling.

### Quantitative reverse transcriptase polymerase chain reaction analysis

Total RNA from each treatment group was used to synthesize cDNA template using the High Capacity cDNA Reverse Transcription kit with random primers (Applied Biosystems, Inc. [ABI], Foster City, CA). Gene-specific primer pairs are listed in Table 1. Amplification was performed on a model 7900HT Fast Real-Time PCR System using SYBR-Green PCRMaster Mix (ABI). Relative quantitation of gene expression between control and experimental treatments was determined using the 18s ribosomal RNA as the endogenous reference. Graphing and statistics were performed using the R statistical environment [107].

### Histological processing, immunocytochemistry, and *in situ* hybridization

Fixation and preparation of embryos for tissue sectioning, immunocytochemistry, and *in situ* hybridization were performed as previously described [19,108,109]. For immunocytochemistry, tissue sections were blocked in 20% goat serum for 30 min, incubated with primary antibody overnight at 4°C, washed in PBS containing 0.0% Triton X-100 (Sigma), then incubated with a fluorescent secondary antibody, and then mounted in VectaShield, with or without DAPI (Vector Labs). Antibodies used were rabbit polyclonal anti-GFP (1:1,000; Torrey Pines Biolabs), chicken anti-GFP (1:1,000; Abcam), mouse monoclonal ZPR-1 which labels double cones (1:200; ZIRC), rabbit polyclonal anti-zebrafish LWS opsin (1:500; gift of David Hyde [80]), FITC- or Cy3-or Alexa-Fluor 647-conjugated secondary antibodies (1:200; Jackson ImmunoResearch). For *in situ* experiments, cRNA probes were generated by *in vitro* reverse transcription of cDNAs. For single *in situ* experiments, digoxigenin- (dig-) UTPs were incorporated into probes for detection with anti-dig antibodies conjugated to alkaline phosphatase and visualized with NBT-BCIP substrate. Dual *in situ* hybridizations were also performed as described [19,110,111]. One probe was generated with dig-UTPs, and the other with fluorescein (FL)-UTPs. Dig probes were detected by anti-dig antibodies conjugated to alkaline phosphatase, followed by the NBT-BCIP phosphatase substrate, resulting in a purple precipitate. Sections were then post-fixed with 4% paraformaldehyde, and the FL probe was detected using anti-FL antibodies conjugated to alkaline phosphatase, followed by the Fast Red phosphatase substrate, resulting in a red fluorescent precipitate [110,112,113].

Probes for *in situs* were generated from the following cDNAs: *LWS1*-specific (3’UTR; [12]); *LWS2*-specific (3’UTR; [12]); *pan-LWS/zfRed* (corresponds directly to *LWS-2* but hybridizes to both *LWS* transcripts; [12,29]), and *Topaz-YFP* (cloned from the *RARE*:*YFP* transgenics [84]).

### Photography and quantification of embryonic *in situ* hybridization patterns

Images were captured using a Leica DMR compound microscope with a SPOT camera system (Diagnostic Instruments). Fluorescently-labeled tissues were viewed using epifluorescence optics, and *in situs* were viewed using epifluorescence, and/or Nomarski (differential interference contrast) optics.

In some experiments, individual, labeled cone photoreceptors were counted within a subset of non-overlapping cryosections. In other experiments, individual, labeled cones were not consistently discernable, and so sections were scored based upon criteria characteristic of *LWS1* or *LWS2* patterns. For *LWS1*: 0 = no expression; 1 = one to five labeled cells in ventral patch; 2 = one-10 in ventral patch, plus one-five scattered beyond ventral retina; 3 = one-10 in ventral patch, plus >5 scattered beyond ventral retina; 4 = widespread label throughout ONL. For *LWS2*: 0 = no expression; 1 = one to five labeled cells in dorsal patch; 2 = less than ½ of dorsal retina strongly labeled, weak label elsewhere; 3 = ½ to ¾ of retina strongly labeled, weaker label elsewhere; 4 = strong label throughout ONL.

Labeling of cones in whole mounts by *in situ* hybridization was quantified using the cone recruitment stages defined in [79], and modified as follows. Stage 0 = no labeling; stage 1 = fewer than 10 cells express the cone opsin; stage 2; 10–20 cones express the cone opsin; stage 3 = more than ¼ but less than 1/3 of the retina expresses the cone opsin; stage 4 = more than 1/3 but less than ½ of the retina expresses the cone opsin; stage 5 = more than ½ but less than the entire retina expresses the cone opsin; stage 6 = entire retina expresses the cone opsin.

### Confocal photography and quantification: *LWS*:*PAC(H)* embryos

*LWS*:*PAC(H)* embryos [82] were maintained in system water with PTU (see above). At 48 hpf, embryos were treated with 9-cis RA, at-RA, or DMSO through 96 hpf (4 dpf) or 108 hpf (4.5 dpf), and then fixed in 4% paraformaldehyde sucrose solution for 1 hour or overnight, washed once in sucrose solution for 30 minutes followed by three washes in PBS. Following fixation and washing, embryos were incubated in PBS at 4°C in the dark for no longer than 24 hours. Immediately prior to imaging, whole eyes were removed from fixed embryos, the sclera teased away by microdissection, and eyes were then coverslipped in glycerol. Imaging was performed at 20X or 60X magnification using an Olympus Fluoview 1000 Laser Scanning confocal microscope running Fluoview ASW software. A z-series covering the entire retinal hemisphere was obtained with 2 micron step sizes. FIJI (ImageJ) was used to flatten z stacks via max projection and adjust brightness/contrast. Images from samples where GFP signal was not resolvable in all planes (due to the developing RPE and/or iridophores in residual sclera) were excluded from analysis.

For pattern analysis, Z stack images of whole mounted eyes acquired from DMSO or 9-cis RA treated *LWS*:*PAC(H)* embryos at 60X magnification were flattened via max projection using FIJI. Rectangular regions with dimension of 40–90 x 40–100 microns were selected from images obtained from samples corresponding to DMSO or 9-cis RA treatment. Cones were identified with an average cell diameter set at 4 μm. Regions were analyzed using WinDRP software (downloaded from the Massaman Lab) to calculate the Nearest Neighbor Distance (NND) and Regularity Index (RI) of the cone mosaic [114,115]. For each region analyzed, patterns were compared against 1000 random simulations of the same number of cells.

### Confocal photography: Juvenile zebrafish sections

One month old juvenile zebrafish were anaesthetized and decapitated. Heads were fixed in 4% paraformaldehyde/sucrose solution as described above for 30 minutes. After 30 minutes, heads were removed and corneas punctured with a dissecting pin, then returned to the fixation solution for another 30 minutes. Heads were then processed for sectioning as previously described [19,108,109] and sectioned at 5 μm. For transgenic *LWS*:*PAC(H)* and/or *RARE*:*YFP* fish, sections were mounted in Vectashield, covered with a coverslip and sealed. Direct imaging of expressed fluorescently tagged proteins was performed at 40X magnification using a Leica DMR compound epifluorescence microscope and SPOT camera, and a NikonAndor spinning disk confocal microscope (water immersion) equipped with a Xyla sCMOS camera. For immunocytochemistry of sections from juvenile fish, sections were stained with antibodies as described above. Imaging was performed at 40X magnification (water immersion) using the Nikon Andor Spinning Disk confocal microscope and Xyla sCMOS camera. Image analysis was performed using FIJI.

## Supporting Information

S1 Table Differential expression of genes in eyes of embryos treated with RA from 48 to 75 hpf. (DOC)

S2 Table Genes differentially expressed in response to RA in zebrafish eyes, whole embryos during somitogenesis, and in heart. (DOCX)

S1 Fig Quantitative PCR for gene expression following treatment with the RXR agonist bexarotene. Box plots indicate relative log2 expression. SciH embryos treated with DMSO (white boxes) or bexarotene (gray boxes) at 0.3 μM (A,B) or 0.06 μM (C,D) for 48–96 hpf were examined for expression of *LWS1* (A,C) and *LWS2* (B,D). In the boxplots, the boxes demarcate the 25^th^ and 75^th^ percentiles, dark horizontal lines designate the medians, and whiskers represent the upper and lower limits. **, p<0.01; 2-tailed Student’s t-test.
(TIF)

S2 Fig Location of predicted retinoic acid response elements (RAREs) on the *LWS* locus on zebrafish chromosome 11. RAREs were identified using TRANSFAC ver. 8.3 and MatInspector. Red bars correspond to sequences 5’-(A/G)GGTCA-3’ [1,2], orange bars to sequences 5’-(A/G)GTTCA-3’ [2], and blue bars to 5’-(A/G)G(G/T)(G/T)(G/A)A-3’ [2]. Bars above vs. below the line refer to sense- vs. antisense-strand directions of these elements. Two potential consensus sites are indicated by asterisks (*) and correspond to 5’-AGGTCA-GG-TGTTCA-3’ (antisense) and 5’AGTTCA-AAA-GGTTCA-3’ (sense) [1]. *LAR*, *LWS* activating region [82].
(TIF)

S1 References References for S2 Fig. (DOCX)
