# Supplementary material for: Retinoic Acid Signaling Regulates Differential Expression of the Tandemly-Duplicated Long Wavelength-Sensitive Cone Opsin Genes in Zebrafish
Source: PLoS Genet. 2015 Aug 21;11(8):e1005483. doi: 10.1371/journal.pgen.1005483 (PMC4546582; doi:10.1371/journal.pgen.1005483)
Supplement: S1 Table — (DOC) [file pgen.1005483.s001.doc]

S1 Table. Differential Expression of Genes in Eyes of Embryos Treated with RA from 48 to 75 hpf

| **#** | **Probe** | **Symbol** | **Description** | | **RMA Expression** | | | | | |
| --- | --- | --- | --- | --- | --- | --- | --- | --- | --- | --- |
|  |  |  |  |  | **Con1** | **Con3** | **Con4** | **RA1** | **RA3** | **RA4** |
| 1 | Dr.180.2.A1_at | cyp26b1 | cytochrome P450, family 26, subfamily b, polypeptide 1 | | 6.14801 | 5.88772 | 6.06676 | 10.3647 | 10.6027 | 10.6797 |
| 2 | Dr.180.1.A1_at | cyp26b1 | cytochrome P450, family 26, subfamily b, polypeptide 1 | | 6.9388 | 6.54291 | 6.86717 | 11.2923 | 11.5047 | 11.3773 |
| 3 | Dr.5757.1.S1_at | cyp26a1 | cytochrome P450, subfamily XXVIA, polypeptide 1 | | 8.60154 | 8.79304 | 8.80153 | 12.3837 | 12.573 | 12.2905 |
| 4 | Dr.5725.1.S1_at | hoxb6b | homeo box B6b | | 3.59407 | 4.02388 | 3.72207 | 7.88922 | 8.00897 | 8.05346 |
| 5 | Dr.26342.1.A1_at | cyp26b1 | cytochrome P450, family 26, subfamily b, polypeptide 1 | | 6.82252 | 6.5821 | 6.73809 | 10.5711 | 10.6547 | 10.3311 |
| 6 | Dr.2644.1.A1_at | dhrs3a | dehydrogenase/reductase (SDR family) member 3a | | 7.5509 | 7.6048 | 7.37408 | 11.2709 | 11.6519 | 11.2688 |
| 7 | Dr.5756.1.S1_at | hoxb5b | homeo box B5b | | 4.08474 | 3.6114 | 3.6901 | 8.72944 | 9.26155 | 9.41649 |
| 8 | Dr.11729.1.S1_at | cyp2j28 | cytochrome P450, family 2, subfamily J, polypeptide 28 | | 4.56883 | 4.30702 | 4.47053 | 6.89268 | 7.02092 | 6.9282 |
| 9 | Dr.8071.1.S1_at | opn1lw1 | opsin 1 (cone pigments), long-wave-sensitive, 1 | | 6.98373 | 6.85 | 7.70418 | 12.1348 | 12.7148 | 12.5121 |
| 10 | Dr.5572.1.S1_at | hoxb5a | homeo box B5a | | 3.1412 | 2.71492 | 2.83891 | 7.79044 | 8.87633 | 8.46274 |
| 11 | Dr.74.1.S1_at | hoxb1b | homeo box B1b | | 5.12907 | 4.7051 | 4.75457 | 8.3434 | 8.90763 | 8.71817 |
| 12 | Dr.12584.1.S1_at | gata3 | GATA-binding protein 3 | | 6.71897 | 6.68771 | 6.49194 | 8.93805 | 9.0957 | 8.89317 |
| 13 | Dr.7232.1.S1_at | hoxb8a | homeo box B8a | | 3.79447 | 3.23316 | 2.89837 | 7.98449 | 8.66662 | 8.71074 |
| 14 | Dr.2693.1.A1_at | |  | | 3.52437 | 3.56909 | 3.52948 | 6.714 | 7.52757 | 6.90419 |
| 15 | DrAffx.2.62.S1_at | cyp26b1 | cytochrome P450, family 26, subfamily b, polypeptide 1 | | 5.27809 | 4.70182 | 4.86196 | 8.3141 | 8.70781 | 9.12421 |
| 16 | Dr.21553.1.A1_at | wu:fc28c04 | wu:fc28c04 | | 2.69675 | 2.77164 | 2.86326 | 5.6631 | 5.79245 | 5.18245 |
| 17 | Dr.5820.1.S1_at | ctsll | cathepsin L, like | | 3.58856 | 3.02256 | 3.14763 | 7.27987 | 7.61912 | 8.5105 |
| 18 | Dr.3529.1.S1_at | hgd | homogentisate 1,2-dioxygenase | | 7.98266 | 7.96773 | 7.69362 | 5.0436 | 5.33745 | 5.58279 |
| 19 | Dr.23911.1.A1_at | slc20a1a | solute carrier family 20, member 1a | | 5.42344 | 5.7188 | 5.24777 | 7.61288 | 7.87825 | 7.72289 |
| 20 | Dr.5727.1.S1_at | hoxb8b | homeo box B8b | | 4.16262 | 3.70524 | 3.83406 | 6.16371 | 6.45463 | 6.60717 |
| 21 | Dr.460.1.A1_at | pvalb2 | parvalbumin 2 | | 10.808 | 10.5786 | 10.5543 | 8.6382 | 8.23258 | 8.6497 |
| 22 | Dr.5723.1.S1_s_at | hoxb8a | homeo box B8a | | 5.10414 | 4.31276 | 4.86704 | 8.78314 | 9.65371 | 10.3443 |
| 23 | Dr.16623.1.A1_at | |  | | 5.68941 | 5.79385 | 5.22521 | 8.03187 | 8.58519 | 8.22626 |
| 24 | Dr.24285.1.A1_at | zgc:158463 | zgc:158463 | | 5.80362 | 5.80244 | 5.86604 | 4.58055 | 4.72866 | 4.64145 |
| 25 | Dr.12378.1.S1_at | hspb1 | heat shock protein, alpha-crystallin-related, 1 | | 7.46533 | 7.73626 | 7.7076 | 9.13268 | 9.12204 | 9.26617 |
| 26 | Dr.26458.1.S1_at | zgc:64022 | zgc:64022 | | 5.3978 | 5.06651 | 5.09062 | 7.05559 | 6.89394 | 6.81086 |
| 27 | Dr.8617.1.A1_at | id:ibd5023 | id:ibd5023 | | 6.82581 | 6.76507 | 6.60802 | 8.65997 | 9.32528 | 8.99674 |
| 28 | Dr.22.1.S1_at | nr2f5 | nuclear receptor subfamily 2, group F, member 5 | | 8.46596 | 8.10252 | 8.32247 | 9.89197 | 9.8439 | 10.0123 |
| 29 | Dr.563.1.S1_at | spon2b | spondin 2b, extracellular matrix protein | | 7.12088 | 6.85563 | 6.31702 | 3.56047 | 4.13935 | 3.98347 |
| 30 | Dr.21414.1.A1_at | hoxb9a | homeo box B9a | | 3.52458 | 3.20836 | 3.29275 | 5.06815 | 5.52965 | 5.26067 |
| 31 | Dr.24899.1.A1_at | wu:fb15e04 | wu:fb15e04 | | 11.4418 | 10.9967 | 11.1284 | 9.3166 | 9.00106 | 8.81653 |
| 32 | Dr.11729.2.S1_a_at | cyp2j28 | cytochrome P450, family 2, subfamily J, polypeptide 28 | | 4.47143 | 4.98475 | 4.78563 | 7.08739 | 7.32777 | 7.88772 |
| 33 | Dr.19483.1.A1_at | zgc:153027 | zgc:153027 | | 5.20776 | 5.05345 | 4.80131 | 6.76 | 6.86664 | 7.16965 |
| 34 | Dr.1192.1.S1_at | ptgds | prostaglandin D2 synthase | | 9.93703 | 10.5244 | 9.85438 | 7.39657 | 7.61693 | 7.88511 |
| 35 | Dr.24233.1.S1_at | fn1b | fibronectin 1b | | 4.45375 | 3.96057 | 3.6662 | 7.90808 | 6.8909 | 7.21014 |
| 36 | Dr.16232.1.A1_at | zgc:77123 | zgc:77123 | | 5.50385 | 5.35622 | 5.45781 | 7.38768 | 7.21525 | 7.84177 |
| 37 | Dr.22681.1.A1_at | wu:fj40c05 | wu:fj40c05 | | 6.15005 | 6.04523 | 5.75199 | 4.21153 | 4.37524 | 4.05076 |
| 38 | Dr.2623.1.A1_at | wu:fc37f07 | wu:fc37f07 | | 8.95629 | 8.7494 | 8.97145 | 7.31506 | 7.42208 | 7.04123 |
| 39 | Dr.13466.1.A1_at | vtnb | vitronectin b | | 5.96679 | 6.15781 | 6.13704 | 7.21707 | 7.29773 | 7.25415 |
| 40 | Dr.642.1.S1_at | myl7 | myosin, light polypeptide 7, regulatory | | 4.57323 | 3.49871 | 3.55854 | 7.02619 | 7.67064 | 7.92581 |
| 41 | Dr.5723.1.A1_at | hoxb8a | homeo box B8a | | 4.66646 | 3.58752 | 3.9448 | 6.92652 | 7.55096 | 7.71653 |
| 42 | Dr.17246.1.A1_at | |  | | 6.31375 | 6.34161 | 5.98932 | 4.5033 | 4.72409 | 4.40179 |
| 43 | Dr.10650.1.A1_at | si:dkey-33i11.4 | si:dkey-33i11.4 | | 6.34546 | 5.88229 | 5.95252 | 7.70774 | 7.76996 | 7.67904 |
| 44 | Dr.24663.1.S1_at | zgc:56565 | zgc:56565 | | 5.78856 | 5.63555 | 5.71418 | 4.65681 | 4.6269 | 4.51113 |
| 45 | Dr.20962.1.S1_at | hoxb2a | homeo box B2a | | 3.04111 | 2.86204 | 2.64751 | 4.4139 | 4.27613 | 4.49428 |
| 46 | Dr.4543.1.S1_at | crygm4 | crystallin, gamma M4 | | 5.05251 | 4.9208 | 4.91707 | 6.71917 | 6.40762 | 6.31004 |
| 47 | Dr.11729.2.A1_at | cyp2j28 | cytochrome P450, family 2, subfamily J, polypeptide 28 | | 3.69725 | 4.58305 | 4.58563 | 6.74274 | 6.9253 | 7.02718 |
| 48 | Dr.15991.1.S1_at | LOC100000332 | similar to ring finger protein 182 | | 5.79237 | 5.4624 | 5.5521 | 7.76492 | 7.59985 | 8.40415 |
| 49 | Dr.5623.1.S1_at | zgc:55345 | zgc:55345 | | 8.73408 | 8.78108 | 8.82993 | 9.75005 | 9.65651 | 9.68888 |
| 50 | Dr.12173.1.S1_at | meis4.1a | myeloid ecotropic viral integration site 4.1a | | 6.87235 | 6.19351 | 6.33377 | 8.42192 | 8.34764 | 8.57788 |
| 51 | Dr.509.1.S1_at | hoxb6a | homeo box B6a | | 4.31791 | 4.20081 | 4.50416 | 6.2992 | 6.96341 | 7.31004 |
| 52 | Dr.23540.1.S1_at | wu:fa99f01 | wu:fa99f01 | | 4.95451 | 5.69236 | 5.39969 | 3.26298 | 3.43518 | 3.40117 |
| 53 | Dr.20928.1.S1_at | pvalb1 | parvalbumin 1 | | 9.09319 | 9.18952 | 9.21395 | 7.74161 | 7.16596 | 7.19355 |
| 54 | Dr.1221.1.A1_at | prrx1b | paired related homeobox 1b | | 8.9777 | 9.02332 | 8.7137 | 7.57896 | 7.39141 | 7.20176 |
| 55 | Dr.10626.1.A1_at | hoxb2a | homeo box B2a | | 3.25419 | 3.06655 | 2.97149 | 4.43519 | 4.27463 | 4.55784 |
| 56 | Dr.11249.1.A1_at | |  | | 4.97173 | 4.5982 | 4.88637 | 7.05869 | 6.56483 | 7.38709 |
| 57 | Dr.12986.1.A1_a_at | fos | v-fos FBJ murine osteosarcoma viral oncogene homolog | | 6.8931 | 6.87809 | 6.25395 | 8.6556 | 8.92938 | 8.5438 |
| 58 | Dr.23086.1.A1_at | wu:fk14d06 | wu:fk14d06 | | 8.20727 | 7.68214 | 8.00152 | 6.12568 | 5.72093 | 6.25821 |
| 59 | Dr.1192.1.S1_a_at | ptgds | prostaglandin D2 synthase | | 8.62935 | 9.51648 | 8.76413 | 6.22028 | 6.56269 | 6.73706 |
| 60 | Dr.18181.1.S1_at | |  | | 8.41917 | 7.76221 | 8.16633 | 9.88954 | 9.83349 | 9.79843 |
| 61 | DrAffx.1.80.S1_at | pvalb4 | parvalbumin 4 | | 8.50877 | 8.38673 | 8.41613 | 6.39091 | 6.78158 | 7.02985 |
| 62 | Dr.10708.1.S1_at | atp1b1b | ATPase, Na+/K+ transporting, beta 1b polypeptide | | 6.5002 | 6.3654 | 6.39434 | 7.85641 | 8.08776 | 8.5078 |
| 63 | Dr.14360.1.A1_at | si:dkey-72l14.3 | si:dkey-72l14.3 | | 7.91026 | 7.92898 | 8.09354 | 9.00125 | 8.91026 | 9.01642 |
| 64 | Dr.24562.1.S1_a_at | mvp | major vault protein | | 6.62807 | 6.801 | 6.40143 | 8.00578 | 8.49835 | 8.41961 |
| 65 | Dr.18181.2.S1_at | |  | | 5.95651 | 5.94812 | 6.65387 | 8.06387 | 8.17517 | 8.36213 |
| 66 | Dr.4697.1.S1_at | col10a1 | collagen, type X, alpha 1 | | 5.64827 | 6.43417 | 5.5166 | 3.69243 | 3.65376 | 3.50606 |
| 67 | Dr.11590.1.A1_s_at | nr0b2a | nuclear receptor subfamily 0, group B, member 2a | | 5.58628 | 5.30734 | 5.12164 | 6.7207 | 6.69471 | 6.70116 |
| 68 | Dr.6063.1.A1_at | si:dkey-73n10.1 | si:dkey-73n10.1 | | 6.51127 | 6.17437 | 5.95465 | 7.73251 | 7.71448 | 7.74004 |
| 69 | Dr.15668.1.S1_at | cryaa | crystallin, alpha A | | 9.78067 | 9.76649 | 10.048 | 11.0439 | 10.9994 | 11.1935 |
| 70 | Dr.5779.1.S1_at | hoxb3a | homeo box B3a | | 4.52367 | 4.24961 | 4.33957 | 5.91858 | 6.68767 | 6.63093 |
| 71 | Dr.4868.1.A1_at | zgc:174888 | zgc:174888 | | 7.50999 | 7.36637 | 7.108 | 8.79513 | 9.43379 | 9.25542 |
| 72 | Dr.13682.1.S1_at | LOC100002387 | hypothetical LOC100002387 | | 8.40769 | 8.32803 | 8.22501 | 7.08873 | 7.2542 | 7.33222 |
| 73 | Dr.12717.1.S1_at | sulf2 | sulfatase 2 | | 6.99014 | 7.29915 | 6.97702 | 5.6688 | 5.46696 | 5.1005 |
| 74 | Dr.3738.1.A1_at | matn4 | matrilin 4 | | 10.4485 | 10.5813 | 10.1326 | 9.02686 | 8.68709 | 8.60333 |
| 75 | Dr.6348.1.S1_at | vil1l | villin 1 like | | 7.80555 | 7.86757 | 7.52404 | 6.38392 | 6.50821 | 6.56512 |
| 76 | Dr.7681.1.A1_at | angptl5 | angiopoietin-like 5 | | 5.99702 | 6.34367 | 5.84006 | 4.6037 | 4.20383 | 4.04051 |
| 77 | Dr.18181.3.S1_at | |  | | 6.98728 | 6.62574 | 7.39883 | 8.85622 | 8.72897 | 9.09854 |
| 78 | Dr.16360.1.A1_at | LOC558079 | similar to dual specificity phosphatase 8 | | 9.44904 | 9.51176 | 9.49109 | 10.2243 | 10.165 | 10.2044 |
| 79 | Dr.9996.1.A1_at | emx2 | empty spiracles homeobox 2 | | 8.53707 | 8.45701 | 8.44454 | 9.60781 | 9.46379 | 9.35304 |
| 80 | Dr.20586.1.A1_at | |  | | 6.55228 | 6.13264 | 5.78127 | 3.62177 | 3.57905 | 4.33602 |
| 81 | Dr.8202.2.S1_a_at | pitx2 | paired-like homeodomain transcription factor 2 | | 8.12345 | 8.30742 | 8.27722 | 9.08368 | 9.17234 | 9.15282 |
| 82 | Dr.10724.1.S1_at | eomesa | eomesodermin homolog a | | 5.53235 | 5.54163 | 5.25477 | 6.85701 | 6.93045 | 6.55417 |
| 83 | Dr.5752.1.S1_at | hoxc5a | homeo box C5a | | 4.2631 | 3.71909 | 3.70179 | 5.43093 | 5.87232 | 6.01392 |
| 84 | Dr.8149.1.A1_at | igfbp2b | insulin-like growth factor binding protein 2b | | 7.17228 | 7.09293 | 7.05748 | 6.31036 | 6.34304 | 6.2093 |
| 85 | Dr.14161.1.S1_at | |  | | 6.30191 | 5.59507 | 5.3966 | 7.60082 | 7.88639 | 8.05375 |
| 86 | Dr.6431.1.S1_at | socs3a | suppressor of cytokine signaling 3a | | 7.35048 | 7.4759 | 6.96723 | 8.57348 | 8.9605 | 8.99866 |
| 87 | Dr.5802.1.S1_at | LOC563410 | similar to transmembrane protein 176B | | 7.72218 | 8.01556 | 7.72505 | 9.04049 | 8.84405 | 8.92911 |
| 88 | Dr.18932.1.A1_at | zgc:153973 | zgc:153973 | | 4.96223 | 4.65286 | 4.40513 | 6.19513 | 6.71353 | 6.98123 |
| 89 | Dr.171.1.A1_at | wu:fc14h11 | wu:fc14h11 | | 7.71614 | 7.72561 | 7.23073 | 9.32909 | 9.06483 | 8.93181 |
| 90 | Dr.5727.1.A1_at | hoxb8b | homeo box B8b | | 4.04772 | 3.73979 | 3.96412 | 4.95196 | 5.29617 | 5.23552 |
| 91 | Dr.8587.1.A2_at | igfbp1a | insulin-like growth factor binding protein 1a | | 8.49302 | 8.55287 | 8.36455 | 10.2034 | 9.59612 | 10.0591 |
| 92 | Dr.5734.1.S1_at | hoxc4a | homeo box C4a | | 3.65593 | 3.2827 | 3.48552 | 4.55229 | 4.6972 | 4.86629 |
| 93 | Dr.11767.1.A1_at | epo | erythropoietin | | 4.99469 | 4.83478 | 5.14754 | 6.72771 | 6.21072 | 6.3031 |
| 94 | Dr.14064.1.S1_at | zgc:55418 | zgc:55418 | | 6.04096 | 6.32913 | 5.86444 | 4.89551 | 4.92242 | 4.81139 |
| 95 | Dr.9617.1.A1_at | socs3b | suppressor of cytokine signaling 3b | | 8.78778 | 8.61149 | 8.49893 | 9.80199 | 10.3676 | 10.4201 |
| 96 | Dr.25534.1.S1_at | sfrp1a | secreted frizzled-related protein 1a | | 6.7679 | 6.4445 | 7.01023 | 8.32033 | 7.99758 | 8.24181 |
| 97 | Dr.11427.1.S1_at | lox | lysyl oxidase | | 5.58322 | 5.44844 | 5.00869 | 6.71187 | 6.69868 | 6.91428 |
| 98 | Dr.18212.1.S1_at | zgc:162651 | zgc:162651 | | 7.79906 | 7.33384 | 7.26236 | 6.01477 | 6.13771 | 5.76506 |
| 99 | Dr.16222.1.A1_at | |  | | 5.3972 | 5.2861 | 4.76595 | 3.50032 | 3.53095 | 3.77176 |
| 100 | Dr.618.1.S1_at | pea3 | ETS-domain transcription factor pea3 | | 7.75204 | 7.661 | 7.57898 | 6.8413 | 6.71998 | 6.86919 |
| 101 | Dr.26486.1.S1_at | six6a | sine oculis-related homeobox 6a | | 7.31513 | 7.00933 | 7.16674 | 8.32038 | 8.36646 | 8.76097 |
| 102 | Dr.3966.1.A1_at | zgc:66052 | zgc:66052 | | 8.98889 | 8.46278 | 7.92813 | 6.51879 | 6.4577 | 6.1785 |
| 103 | Dr.10713.1.S1_at | atp1a1a.3 | ATPase, Na+/K+ transporting, alpha 1a.3 polypeptide | | 5.76034 | 5.87509 | 5.24226 | 7.43706 | 7.01773 | 7.30336 |
| 104 | Dr.4119.2.S1_at | hoxd4a | homeo box D4a | | 4.15701 | 3.35479 | 3.52481 | 5.92656 | 5.87316 | 5.29682 |
| 105 | Dr.25653.1.A1_at | zic3 | zic family member 3 heterotaxy 1 (odd-paired homolog, Drosophila) | | 8.41679 | 8.06982 | 8.14425 | 7.01957 | 7.22852 | 7.12857 |
| 106 | Dr.24774.1.S2_at | hoxa4a | homeo box A4a | | 3.80406 | 3.37742 | 3.18785 | 5.75126 | 5.77567 | 4.94183 |
| 107 | Dr.23439.2.S1_s_at | zgc:111913 | zgc:111913 | | 8.48353 | 8.15098 | 7.73626 | 6.37032 | 6.09478 | 6.65416 |
| 108 | Dr.3981.1.A1_at | |  | | 7.6187 | 7.53081 | 7.20749 | 8.53865 | 8.5006 | 8.54263 |
| 109 | Dr.16109.1.S1_at | ror2 | receptor tyrosine kinase-like orphan receptor 2 | | 6.48075 | 6.55599 | 6.5744 | 7.58858 | 7.88262 | 7.44015 |
| 110 | Dr.23386.1.A1_at | LOC557223 | similar to F59A2.6 | | 5.94957 | 5.67456 | 6.06697 | 4.79115 | 4.30781 | 4.61684 |
| 111 | Dr.8587.1.A1_at | igfbp1a | insulin-like growth factor binding protein 1a | | 8.34926 | 8.13264 | 7.84102 | 9.88883 | 9.44042 | 10.2619 |
| 112 | Dr.12949.1.S1_at | dio2 | deiodinase, iodothyronine, type II | | 4.27983 | 4.70257 | 4.50356 | 5.5355 | 5.61717 | 5.82108 |
| 113 | Dr.18756.1.S1_at | |  | | 4.52354 | 4.23563 | 3.96244 | 5.47258 | 5.48558 | 5.78244 |
| 114 | Dr.14082.1.A1_at | |  | | 4.88364 | 5.01568 | 5.17311 | 6.08522 | 5.85775 | 6.03777 |
| 115 | Dr.5206.1.S1_at | aldh1a2 | aldehyde dehydrogenase 1 family, member A2 | | 7.48121 | 7.02901 | 6.99054 | 5.93952 | 5.64152 | 5.33177 |
| 116 | Dr.17511.1.S1_at | cyp2j25 | cytochrome P450, family 2, subfamily J, polypeptide 25 | | 4.77496 | 4.59368 | 4.68221 | 5.46421 | 5.45868 | 5.37611 |
| 117 | Dr.4314.1.A1_a_at | wu:fb95d03 | wu:fb95d03 | | 7.66388 | 7.47383 | 6.93254 | 8.83013 | 8.82571 | 8.75462 |
| 118 | Dr.20010.12.S1_at | hapln1a | hyaluronan and proteoglycan link protein 1a | | 4.13505 | 4.4614 | 4.56025 | 3.39729 | 3.08048 | 3.15958 |
| 119 | Dr.12986.1.A1_at | fos | v-fos FBJ murine osteosarcoma viral oncogene homolog | | 6.27371 | 5.98868 | 5.71862 | 7.70251 | 8.12527 | 7.29096 |
| 120 | Dr.1131.1.A1_at | zgc:110340 | zgc:110340 | | 9.31327 | 8.93013 | 9.09614 | 10.2693 | 10.0081 | 10.1861 |
| 121 | Dr.20010.16.A1_at | hapln1a | hyaluronan and proteoglycan link protein 1a | | 6.05697 | 6.09226 | 6.14967 | 5.18803 | 5.05269 | 4.67626 |
| 122 | Dr.4321.1.A1_at | zgc:136858 | zgc:136858 | | 3.38453 | 3.3896 | 3.52897 | 2.74839 | 2.66608 | 2.76603 |
| 123 | Dr.22517.1.S1_at | si:dkey-127j5.5 | si:dkey-127j5.5 | | 9.40034 | 9.32965 | 9.34533 | 10.4891 | 10.4033 | 10.1019 |
| 124 | Dr.10376.1.S1_at | |  | | 6.1429 | 5.84299 | 5.54497 | 6.99613 | 7.34381 | 7.13248 |
| 125 | Dr.4314.1.A1_x_at | wu:fb95d03 | wu:fb95d03 | | 5.85354 | 6.0379 | 5.56824 | 6.84641 | 6.90555 | 7.10471 |
| 126 | Dr.567.2.S1_a_at | bmp4 | bone morphogenetic protein 4 | | 5.03202 | 4.94926 | 5.0202 | 4.4041 | 4.38633 | 4.30584 |
| 127 | Dr.22588.1.A1_at | wu:fj19a05 | wu:fj19a05 | | 5.43575 | 5.72748 | 6.12278 | 4.32625 | 3.76942 | 4.35227 |
| 128 | Dr.6437.1.S1_at | ptpn6 | protein tyrosine phosphatase, non-receptor type 6 | | 6.17549 | 5.9595 | 6.24101 | 7.02245 | 7.533 | 7.43059 |
| 129 | Dr.21227.1.A1_at | zgc:66097 | zgc:66097 | | 7.07071 | 6.61322 | 6.70413 | 5.67539 | 5.3417 | 5.71169 |
| 130 | Dr.23423.2.S1_s_at | zp2.3 | zona pellucida glycoprotein 2.3 | | 7.21752 | 7.0023 | 7.09028 | 5.24279 | 5.24553 | 6.05582 |
| 131 | Dr.8142.1.S1_at | aanat2 | arylalkylamine N-acetyltransferase | | 6.75645 | 6.70016 | 6.79829 | 7.69218 | 8.3456 | 8.4462 |
| 132 | AFFX-Dr-NM_131175-1_s_at | | | | 9.17383 | 9.62415 | 8.88473 | 10.4523 | 10.7003 | 10.6127 |
| 133 | Dr.25653.2.S1_at | zic3 | | zic family member 3 heterotaxy 1 (odd-paired homolog, Drosophila) | 6.78032 | 6.52608 | 6.365 | 5.54723 | 5.59361 | 5.67187 |
| 134 | Dr.17008.1.A1_at | hnrnpa0 | | heterogeneous nuclear ribonucleoprotein A0 | 7.77455 | 7.99486 | 7.75998 | 6.66692 | 7.06255 | 6.73355 |
| 135 | Dr.322.2.A1_a_at | | |  | 6.90313 | 6.92355 | 6.64678 | 5.97431 | 6.04918 | 6.01586 |
| 136 | Dr.7888.1.S1_at | rrs1 | | RRS1 ribosome biogenesis regulator homolog (S. cerevisiae) | 7.7004 | 7.46038 | 7.89629 | 6.75875 | 6.73859 | 6.69703 |
| 137 | Dr.4907.1.S1_at | fgg | | fibrinogen, gamma polypeptide | 4.7366 | 4.0141 | 4.03148 | 6.66803 | 9.43148 | 9.41268 |
| 138 | Dr.4603.1.A1_at | nrip1b | | nuclear receptor interacting protein 1b | 8.44533 | 8.39826 | 8.4655 | 9.08054 | 9.00899 | 8.97775 |
| 139 | Dr.9926.1.S1_at | zgc:64006 | | zgc:64006 | 7.7265 | 7.8802 | 7.59708 | 6.49472 | 6.84597 | 6.84165 |
| 140 | Dr.7608.2.S1_at | jun | | v-jun sarcoma virus 17 oncogene homolog (avian) | 7.44024 | 7.57753 | 7.68462 | 8.32003 | 8.61748 | 8.70599 |
| 141 | Dr.26207.1.A1_at | inhbaa | | inhibin, beta Aa | 7.83682 | 7.87547 | 7.40806 | 6.69674 | 6.1908 | 6.43776 |
| 142 | Dr.2377.1.A1_at | krt1-19d | | keratin, type 1, gene 19d | 7.19567 | 6.74698 | 7.31679 | 6.0065 | 5.84547 | 5.964 |
| 143 | Dr.8056.1.S1_at | dkk1 | | dickkopf 1 | 6.25882 | 6.54028 | 6.56322 | 5.67059 | 5.55539 | 5.60579 |
| 144 | Dr.19643.1.A1_at | | |  | 8.51525 | 8.56655 | 8.15239 | 7.34435 | 6.49879 | 6.67088 |
| 145 | Dr.5853.1.A1_at | lgals3bpb | | lectin, galactoside-binding, soluble, 3 binding protein b | 6.57594 | 7.37002 | 6.78177 | 8.31031 | 8.74535 | 8.28989 |
| 146 | Dr.1999.1.S1_at | zgc:109940 | | zgc:109940 | 6.55701 | 5.44383 | 6.27973 | 7.71857 | 8.17908 | 8.56665 |
| 147 | Dr.3613.1.S1_at | cp | | ceruloplasmin | 5.90702 | 5.72925 | 5.36372 | 6.65285 | 6.80658 | 6.84052 |
| 148 | Dr.11135.1.S1_at | | |  | 7.48275 | 7.41188 | 7.28739 | 8.22772 | 8.64031 | 8.27799 |
| 149 | Dr.8192.1.S1_at | hoxc1a | | homeo box C1a | 5.49597 | 5.21649 | 5.04281 | 6.17513 | 6.51722 | 6.67494 |
| 150 | Dr.7608.1.A1_at | jun | | v-jun sarcoma virus 17 oncogene homolog (avian) | 8.28201 | 8.58558 | 8.23731 | 9.51257 | 9.65349 | 9.20103 |
| 151 | Dr.20994.1.S1_s_at | meis4.1a | | myeloid ecotropic viral integration site 4.1a | 4.66452 | 4.7756 | 5.11521 | 6.05844 | 6.1542 | 6.8428 |
| 152 | Dr.23002.1.A1_at | | |  | 5.79646 | 5.7256 | 5.61036 | 6.59649 | 6.34091 | 6.55514 |
| 153 | Dr.12401.1.S1_at | epha4b | | eph receptor A4b | 4.4629 | 5.15024 | 4.9952 | 5.95719 | 6.34203 | 6.33368 |
| 154 | Dr.2499.1.S1_at | myog | | myogenin | 5.6967 | 6.22988 | 5.82852 | 6.9292 | 7.15284 | 6.93117 |
| 155 | Dr.7599.1.A1_at | LOC100149143 | | similar to toxin-1 | 7.07946 | 7.32564 | 6.64888 | 8.06088 | 8.49321 | 8.43054 |
| 156 | Dr.14169.1.A1_at | slc25a32a | | solute carrier family 25, member 32a | 10.351 | 10.3937 | 10.2588 | 10.9901 | 10.9011 | 11.0796 |
| 157 | Dr.12308.1.S1_at | irf9 | | interferon regulatory factor 9 | 5.24338 | 5.19041 | 5.22683 | 6.03839 | 6.77714 | 6.48477 |
| 158 | Dr.16174.1.A1_at | si:dkey-201c13.3 | | si:dkey-201c13.3 | 8.17705 | 8.81792 | 8.1832 | 9.54873 | 9.57831 | 9.78236 |
| 159 | Dr.4938.1.S1_at | fads2 | | fatty acid desaturase 2 | 7.79789 | 7.71947 | 7.74314 | 8.48772 | 8.28716 | 8.41285 |
| 160 | Dr.21127.1.A1_at | wu:fb78c07 | | wu:fb78c07 | 6.05238 | 6.07724 | 6.23491 | 7.63253 | 7.8753 | 7.01026 |
| 161 | Dr.8242.1.A1_at | vim | | vimentin | 8.39988 | 8.65931 | 8.57547 | 9.33506 | 9.80515 | 9.95225 |
| 162 | Dr.26003.1.A1_at | bbc3 | | BCL2 binding component 3 | 6.89201 | 7.07007 | 7.09448 | 8.31586 | 7.90513 | 7.83784 |
| 163 | Dr.16163.1.S1_at | bcor | | BCL6 co-repressor | 7.53751 | 7.7218 | 7.4858 | 8.24838 | 8.32823 | 8.23074 |
| 164 | Dr.462.1.A1_at | wu:fa05a04 | | wu:fa05a04 | 2.97009 | 3.04712 | 3.0186 | 4.45531 | 4.72616 | 3.86054 |
| 165 | Dr.15554.1.A1_at | si:ch211-237l4.5 | | si:ch211-237l4.5 | 7.90963 | 7.55796 | 7.33116 | 8.54054 | 8.88671 | 8.71047 |
| 166 | Dr.11590.1.A1_at | nr0b2a | | nuclear receptor subfamily 0, group B, member 2a | 6.42189 | 5.88804 | 5.69674 | 7.32972 | 7.2442 | 7.10148 |
| 167 | Dr.9217.1.A1_at | | |  | 8.08935 | 8.61814 | 8.58845 | 9.49033 | 9.69321 | 9.39981 |
| 168 | Dr.16677.1.A1_at | LOC795739 | | similar to zinc fingers and homeoboxes 3 | 4.8408 | 4.75147 | 4.69846 | 5.3133 | 5.40357 | 5.49979 |
| 169 | Dr.8100.1.S1_at | gata5 | | GATA-binding protein 5 | 3.52569 | 3.23227 | 3.20792 | 4.59569 | 5.37103 | 4.45219 |
| 170 | Dr.20027.1.S1_at | znf703 | | zinc finger protein 703 | 8.34697 | 8.76067 | 8.26105 | 9.4095 | 9.62942 | 9.37958 |
| 171 | Dr.4615.2.S1_at | | |  | 6.17952 | 6.53521 | 6.70593 | 7.35114 | 7.48371 | 7.62479 |
| 172 | Dr.348.1.S1_at | rxrga | | retinoid x receptor, gamma a | 7.75743 | 7.69751 | 7.66965 | 8.21847 | 8.42678 | 8.35357 |
| 173 | Dr.568.1.S1_at | bmp2b | | bone morphogenetic protein 2b | 6.24498 | 6.19246 | 6.26806 | 6.69467 | 6.70959 | 6.71925 |
| 174 | Dr.6568.1.A1_at | wu:fj41a03 | | wu:fj41a03 | 8.70087 | 8.28671 | 8.38579 | 9.33521 | 9.27858 | 9.26576 |
