# Supplementary material for: Retinoic Acid Signaling Regulates Differential Expression of the Tandemly-Duplicated Long Wavelength-Sensitive Cone Opsin Genes in Zebrafish
Source: PLoS Genet. 2015 Aug 21;11(8):e1005483. doi: 10.1371/journal.pgen.1005483 (PMC4546582; doi:10.1371/journal.pgen.1005483)
Supplement: S2 Table — (DOCX) [file pgen.1005483.s002.docx]

S2 Table. Genes Differentially Expressed in Response to RA in Zebrafish Eyes, Whole Embryos during Somitogenesis, and in Heart.

| **#** | **Probe** | **Symbol** | **Description** | **Log2 Fold Change** | | |
| --- | --- | --- | --- | --- | --- | --- |
|  |  |  |  | **Eyes** | **Whole embryo somito-genesis** | **Heart** |
| 1 | Dr.180.2.A1_at |  |  | 4.52 | 0.465 | 1.75 |
| 2 | Dr.180.1.A1_at | cyp26b1 | cytochrome P450, family 26, subfamily b, polypeptide 1 | 4.61 | 0.705 | 3.58 |
| 3 | Dr.5757.1.S1_at | cyp26a1 | cytochrome P450, subfamily XXVIA, polypeptide 1 | 3.68 | 2.86 | 6.71 |
| 4 | Dr.5725.1.S1_at | hoxb6b | homeo box B6b | 4.2 | 2.3 | Not DE^1^ |
| 5 | Dr.26342.1.A1_at | cyp26b1 | cytochrome P450, family 26, subfamily b, polypeptide 1 | 4.2 | Not DE | 3.61 |
| 6 | Dr.2644.1.A1_at | dhrs3a | dehydrogenase/reductase (SDR family) member 3a | 3.89 | 3.14 | 5.99 |
| 7 | Dr.5756.1.S1_at | hoxb5b | homeo box B5b | 5.34 | 3.69 | Not DE |
| 8 | Dr.5572.1.S1_at | hoxb5a | homeo box B5a | 5.48 | 4.25 | Not DE |
| 9 | Dr.74.1.S1_at | hoxb1b | homeo box B1b | 5.48 | 1.92 | Not DE |
| 10 | Dr.7232.1.S1_at | hoxb8a | homeo box B8a | 5.15 | 1.52 | Not DE |
| 11 | Dr.2693.1.A1_at | hoxb8a | homeo box B8a | 5.15 | 1.56 | Not DE |
| 12 | DrAffx.2.62.S1_at | cyp26b1 | cytochrome P450, family 26, subfamily b, polypeptide 1 | 0.234 | 0.546 | 4.38 |
| 13 | Dr.5727.1.S1_at | hoxb8b | homeo box B8b | 0.13 | 1.32 | Not DE |
| 14 | Dr.5723.1.S1_s_at | hoxb8b | homeo box B8b | 2.51 | 1.48 | Not DE |
| 15 | Dr.16623.1.A1_at |  |  | 2.71 | 0.496 | 2.88 |
| 16 | Dr.8617.1.A1_at | hapln1b | hyaluronan and proteoglycan link protein 1b | 2.26 | 1.03 | Not DE |
| 17 | Dr.22.1.S1_at | nr2f5 | nuclear receptor subfamily 2, group F, member 5 | 1.62 | 0.475 | 3.09 |
| 18 | Dr.1192.1.S1_at | ptgds | prostaglandin D2 synthase | -2.47 | -1.87 | Not DE |
| 19 | Dr.2623.1.A1_at |  |  | -1.63 | 2.8 | Not DE |
| 20 | Dr.5723.1.A1_at | hoxb8a | homeo box B8a | 3.34 | 0.356 | Not DE |
| 21 | Dr.20962.1.S1_at | hoxb2a | homeo box B2a | 1.55 | 1.45 | Not DE |
| 22 | Dr.12173.1.S1_at | meis4.1a | myeloid ecotropic viral integration site 4.1a | 1.98 | 0.774 | Not DE |
| 23 | Dr.509.1.S1_at | hoxb6a | homeo box B6a | 2.52 | 1.37 | Not DE |
| 24 | Dr.10626.1.A1_at | hoxb2a | homeo box B2a | 1.33 | 1.3 | Not DE |
| 25 | Dr.1192.1.S1_a_at |  |  | -2.47 | -1.85 | Not DE |
| 26 | Dr.11590.1.A1_s_at | nr0b2a | nuclear receptor subfamily 0, group B, member 2a | 1.37 | 0.832 | 2.39 |
| 27 | Dr.5779.1.S1_at | hoxb3a | homeo box B3a | 2.04 | 1.82 | Not DE |
| 28 | Dr.18181.3.S1_at |  |  | 1.89 | 0.705 | Not DE |
| 29 | Dr.8202.2.S1_a_at | pitx2 | paired-like homeodomain transcription factor 2 | 0.903 | -1.26 | Not DE |
| 30 | Dr.5734.1.S1_at | hoxc4a | homeo box C4a | 1.23 | 1.71 | Not DE |
| 31 | Dr.3966.1.A1_at | zgc:66052 | zgc:66052 | -2.08 | -0.813 | Not DE |
| 32 | Dr.4119.2.S1_at | hoxd4a | homeo box D4a | 2.2 | 2.34 | Not DE |
| 33 | Dr.25653.1.A1_at | zic3 | zic family member 3 heterotaxy 1 (odd-paired homolog, Drosophila) | -1.09 | -0.783 | Not DE |
| 34 | Dr.24774.1.S2_at |  |  | 2.03 | 2.8 | Not DE |
| 35 | Dr.16109.1.S1_at |  |  | 1.1 | 0.454 | Not DE |
| 36 | Dr.18756.1.S1_at |  |  | 1.34 | 0.864 | Not DE |
| 37 | Dr.14082.1.A1_at |  |  | 0.971 | 1.12 | Not DE |
| 38 | Dr.5206.1.S1_at | aldh1a2 | aldehyde dehydrogenase 1 family, member A2 | -1.53 | -0.986 | Not DE |
| 39 | Dr.4314.1.A1_a_at |  |  | 1.45 | 0.978 | 1.48 |
| 40 | Dr.4314.1.A1_x_at |  |  | 1.13 | 0.934 | Not DE |
| 41 | Dr.22588.1.A1_at | wu:fj19a05 | wu:fj19a05 | -1.61 | 0.782 | Not DE |
| 42 | Dr.25653.2.S1_at | zic3 | zic family member 3 heterotaxy 1 (odd-paired homolog, Drosophila) | -0.952 | -0.844 | Not DE |
| 43 | Dr.4907.1.S1_at | fgg | fibrinogen, gamma polypeptide | 4.24 | 3.38 | Not DE |
| 44 | Dr.4603.1.A1_at | nrip1b | nuclear receptor interacting protein 1b | 0.585 | 1.67 | Not DE |
| 45 | Dr.8192.1.S1_at | hoxc1a | homeo box C1a | 1.2 | 1.77 | Not DE |
| 46 | Dr.20994.1.S1_s_at | meis4.1a | myeloid ecotropic viral integration site 4.1a | 1.5 | 0.872 | Not DE |
| 47 | Dr.21127.1.A1_at | wu:fb78c07 | wu:fb78c07 | 1.38 | 0.496 | Not DE |
| 48 | Dr.11590.1.A1_at | nr0b2a | nuclear receptor subfamily 0, group B, member 2a | 1.22 | 0.84 | 2.39 |
| 49 | Dr.20027.1.S1_at | znf703 | zinc finger protein 703 | 1.01 | 0.911 | Not DE |
| 50 | Dr.348.1.S1_at | rxrga | retinoid x receptor, gamma a | 0.623 | 2.4 | Not DE |
| 51 | Dr.568.1.S1_at | bmp2b | bone morphogenetic protein 2b | 0.475 | -0.606 | Not DE |

^1^ not DE, not differentially expressed.
