# Supplementary figures and images for: Retinoic Acid Signaling Regulates Differential Expression of the Tandemly-Duplicated Long Wavelength-Sensitive Cone Opsin Genes in Zebrafish
Source: PLoS Genet. 2015 Aug 21;11(8):e1005483. doi: 10.1371/journal.pgen.1005483 (PMC4546582; doi:10.1371/journal.pgen.1005483)

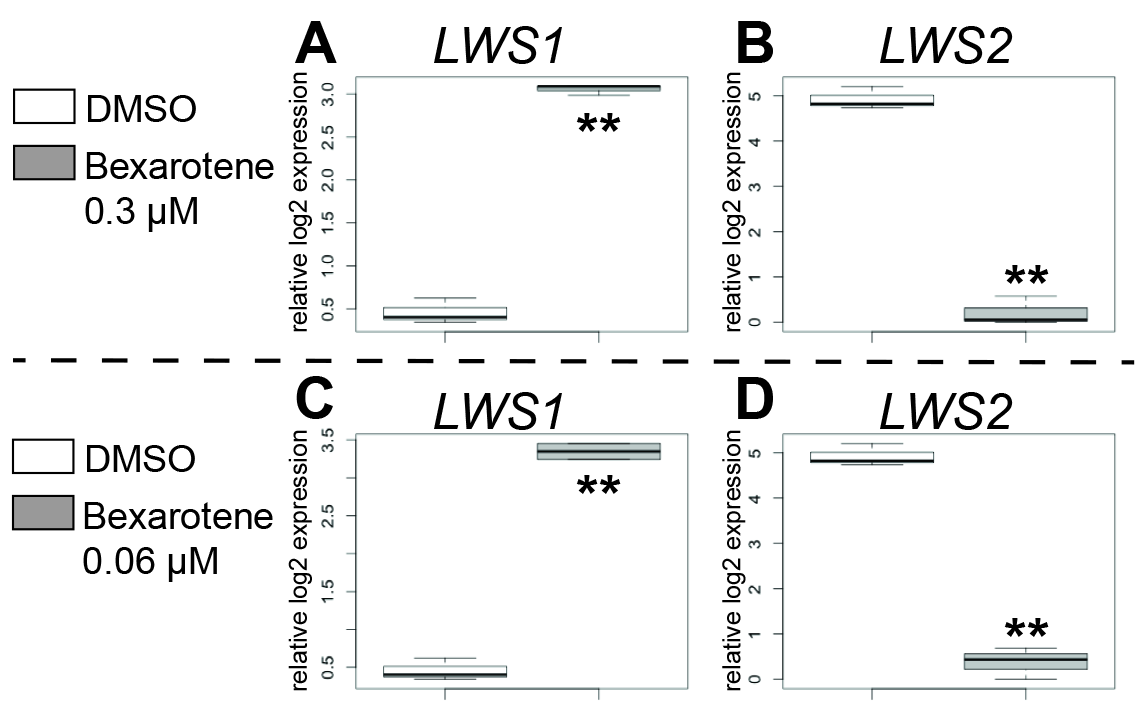

Supplement: S1 Fig — Box plots indicate relative log2 expression. SciH embryos treated with DMSO (white boxes) or bexarotene (gray boxes) at 0.3 μM (A,B) or 0.06 μM (C,D) for 48–96 hpf were examined for expression of LWS1 (A,C) and LWS2 (B,D). In the boxplots, the boxes demarcate the 25th and 75th percentiles, dark horizontal lines designate the medians, and whiskers represent the upper and lower limits. **, p<0.01; 2-tailed Student’s t-test. (TIF) [file pgen.1005483.s003.tif]

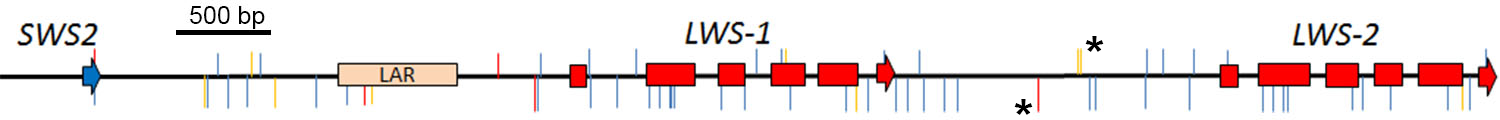

Supplement: S2 Fig — RAREs were identified using TRANSFAC ver. 8.3 and MatInspector. Red bars correspond to sequences 5’-(A/G)GGTCA-3’ [1,2], orange bars to sequences 5’-(A/G)GTTCA-3’ [2], and blue bars to 5’-(A/G)G(G/T)(G/T)(G/A)A-3’ [2]. Bars above vs. below the line refer to sense- vs. antisense-strand directions of these elements. Two potential consensus sites are indicated by asterisks (*) and correspond to 5’-AGGTCA-GG-TGTTCA-3’ (antisense) and 5’AGTTCA-AAA-GGTTCA-3’ (sense) [1]. LAR, LWS activating region [82]. (TIF) [file pgen.1005483.s004.tif]
