# Supplementary material for: Retinoic Acid Signaling Regulates Differential Expression of the Tandemly-Duplicated Long Wavelength-Sensitive Cone Opsin Genes in Zebrafish
Source: PLoS Genet. 2015 Aug 21;11(8):e1005483. doi: 10.1371/journal.pgen.1005483 (PMC4546582; doi:10.1371/journal.pgen.1005483)
Supplement: S1 References — (DOCX) [file pgen.1005483.s005.docx]

S2 Fig References.

1. Linney E, Donerly S, Mackey L, Dobbs-McAuliffe B (2011) The negative side of retinoic acid receptors. Neurotoxicol Teratol 33: 631-640.

2. Balmer JE, Blomhoff R (2002) Gene expression regulation by retinoic acid. J Lipid Res 43: 1773-1808.
